# Supplementary figures and images for: Negative linkage disequilibrium between amino acid changing variants reveals interference among deleterious mutations in the human genome
Source: PLoS Genet. 2021 Jul 28;17(7):e1009676. doi: 10.1371/journal.pgen.1009676 (PMC8351996; doi:10.1371/journal.pgen.1009676)

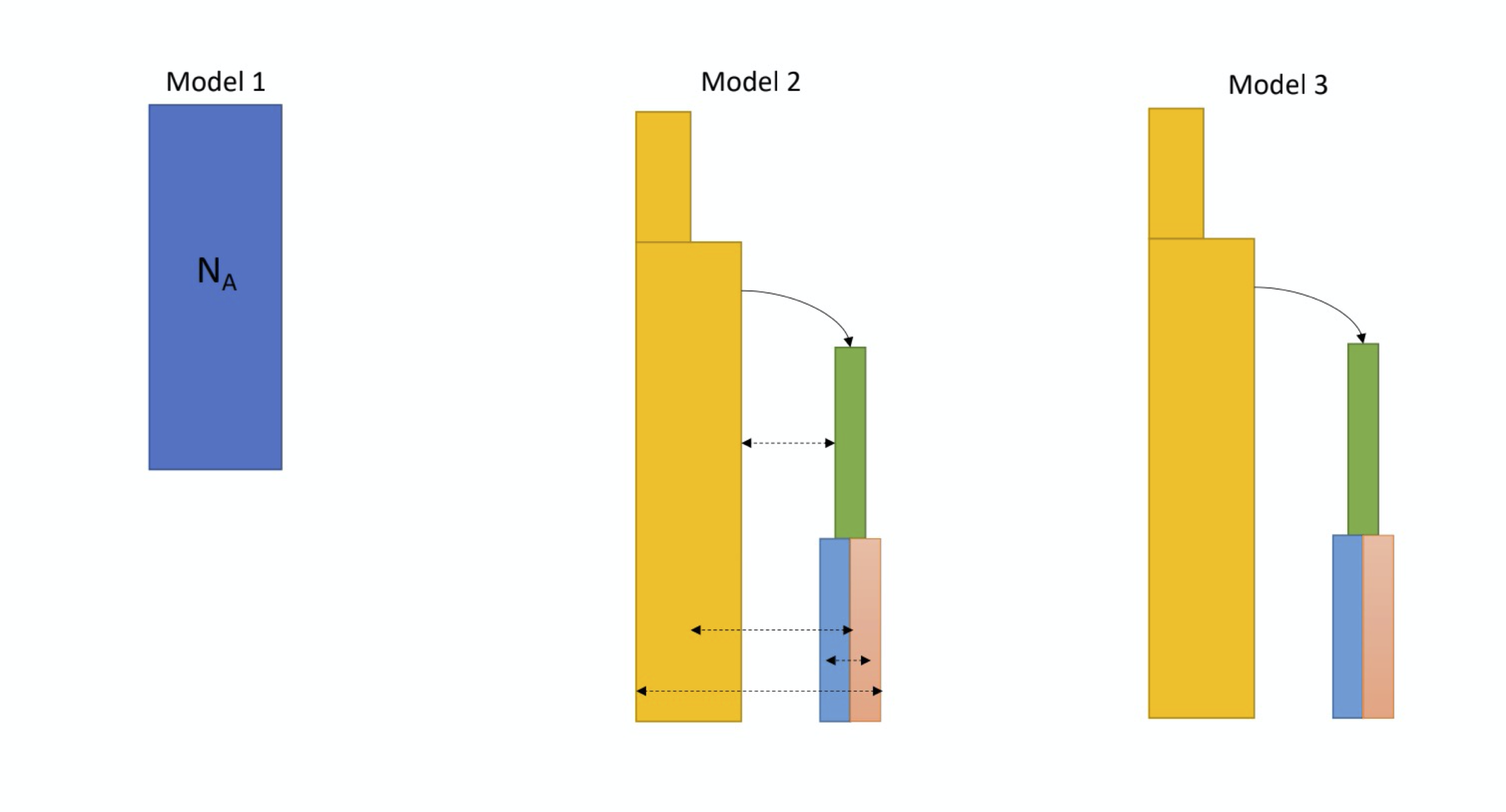

Supplement: S1 Fig — Model 1 represents a constant population size simulation. Model 2 represents the Gravel et. al (2011) demographic model. The yellow subpopulation represents Africans (YRI). Green represents the ancestral Eurasian bottleneck. Blue represents East Asia and pink represents Europe. Model 3 is the Gravel demographic model but without migration. (TIFF) [file pgen.1009676.s001.tiff]

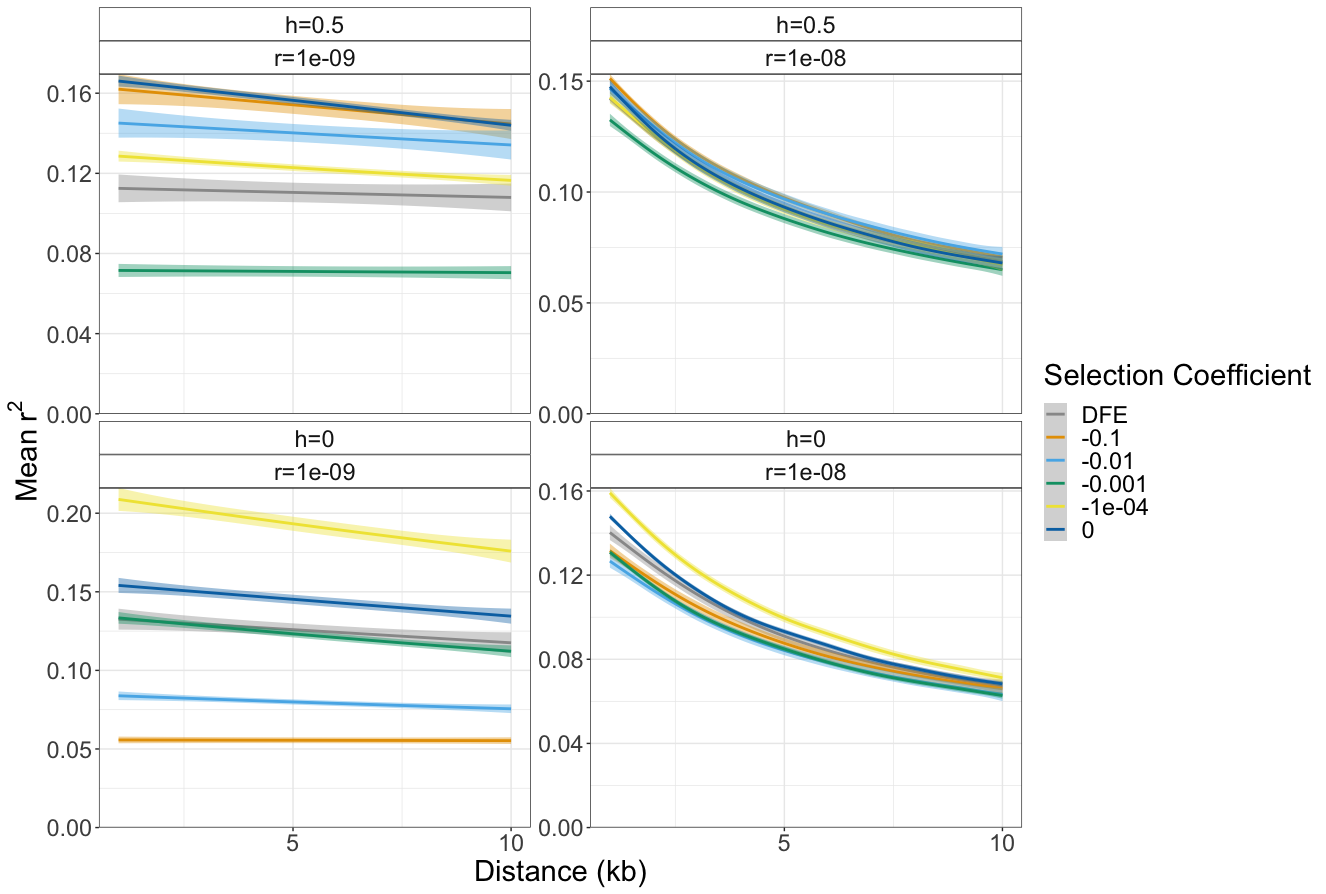

Supplement: S2 Fig — The differences in the LD decay curves are most apparent with recombination rate r = 1 x 10^−9^ per bp and depend on the dominance coefficient (h) of mutations and the selection coefficient (s) of NS mutations. DFE curves come from simulated populations where new NS mutations have selection coefficients following our defined distribution of fitness effects (see text). All variants are included in this analysis and no frequency filters are applied. (TIFF) [file pgen.1009676.s002.tiff]

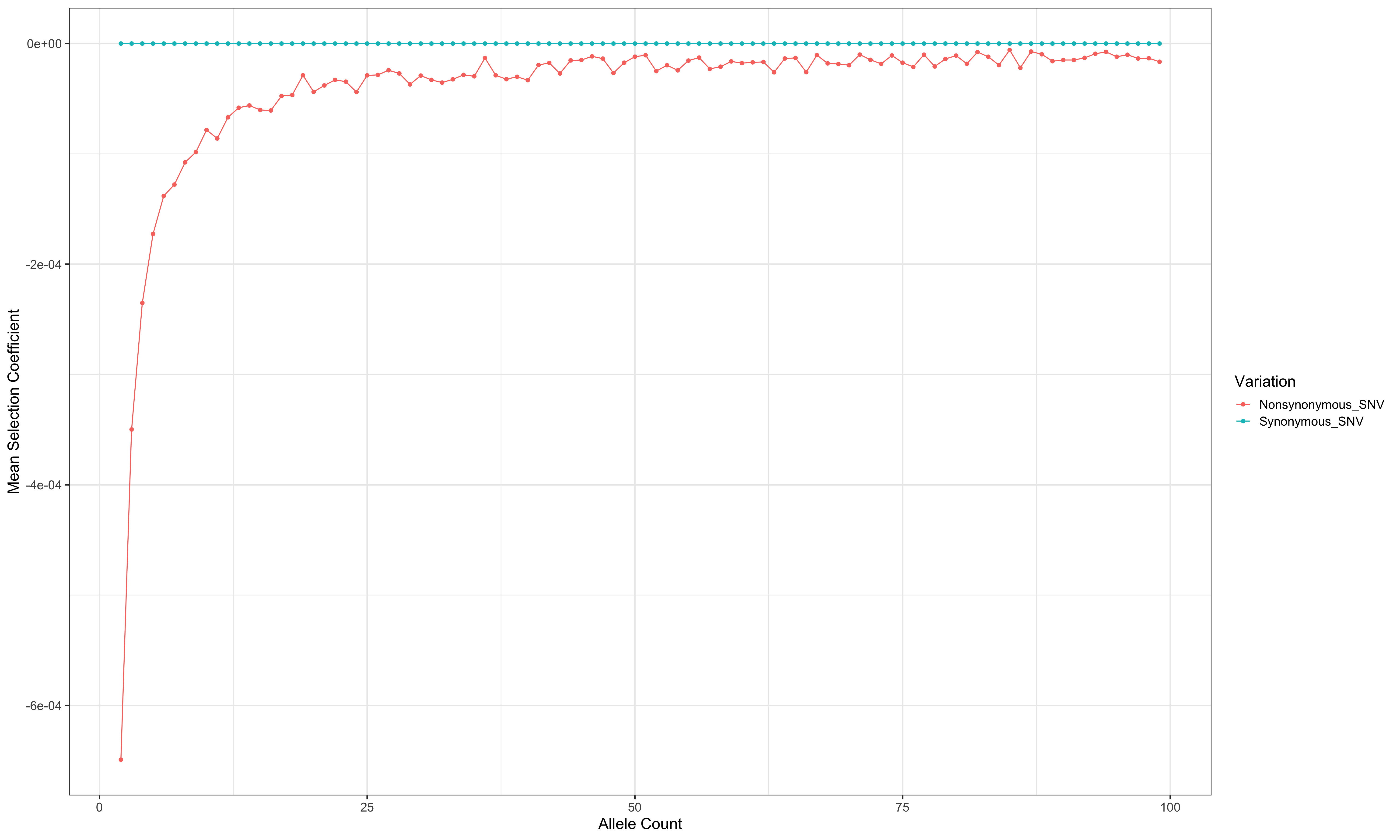

Supplement: S3 Fig — We simulated 300 replicates of a constant population size of 14, 474 diploid individuals with r = 1 x 10^−8^ per bp, and the DFE and genome structure defined in Materials and Methods. Although simulations predict singletons (first red point), on average, should be the most deleterious in our samples, doubletons (second red point) are also relatively deleterious. Higher frequency variants tend to have mean selection coefficients that are much more neutral. Because we want to study the effects of negative selection on LD, we restrict many of our analyses to low frequency variants (allele count < = 5) (TIFF) [file pgen.1009676.s003.tiff]

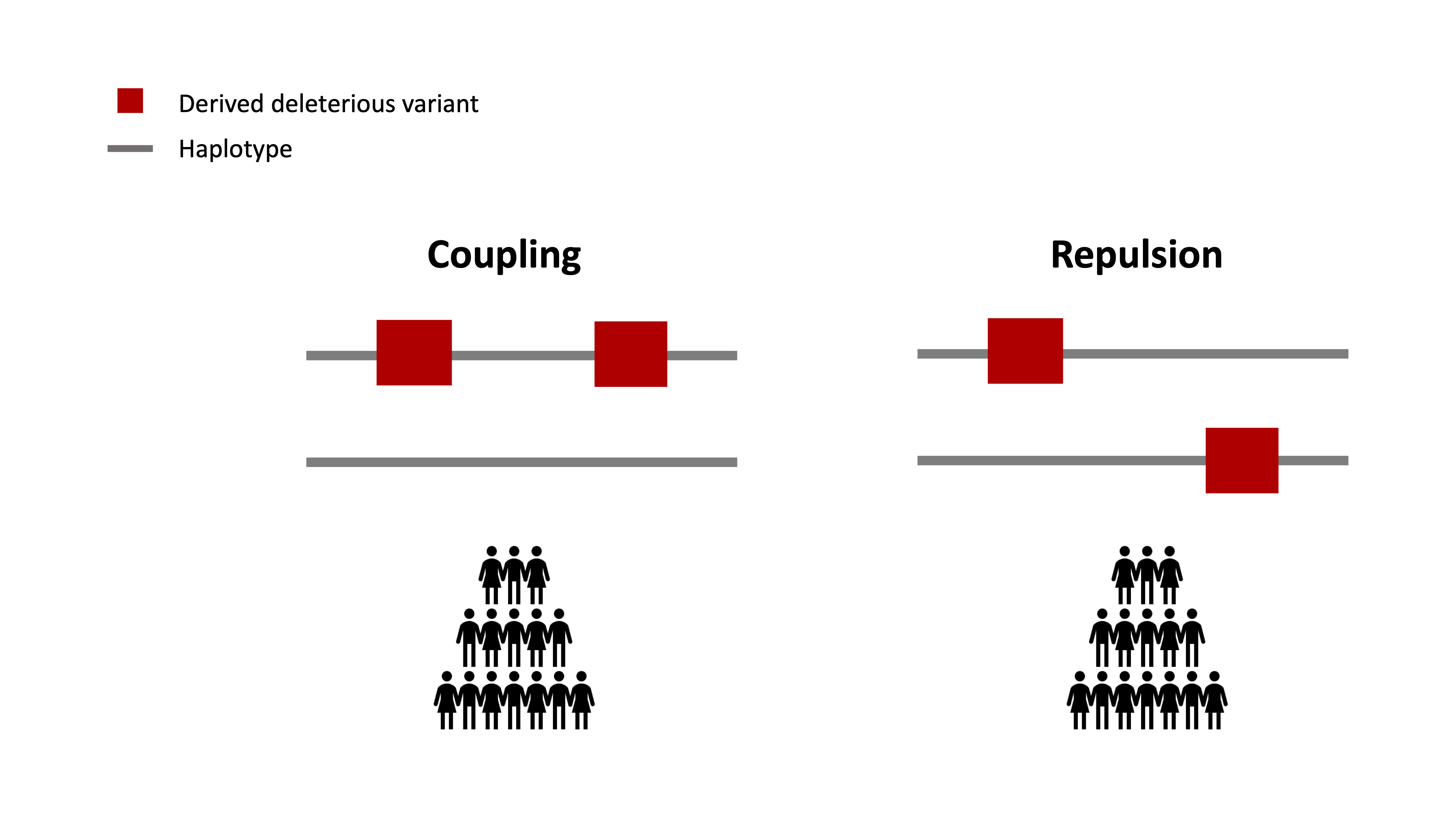

Supplement: S4 Fig — Pairs of derived variants that co-occur on the same haplotypes more frequently than expected are said to be “coupling” or in positive LD (left panel). Pairs of derived variants that occur less frequently than expected on the same haplotype are said to be in “repulsion” or in negative LD (right panel). (TIFF) [file pgen.1009676.s004.tiff]

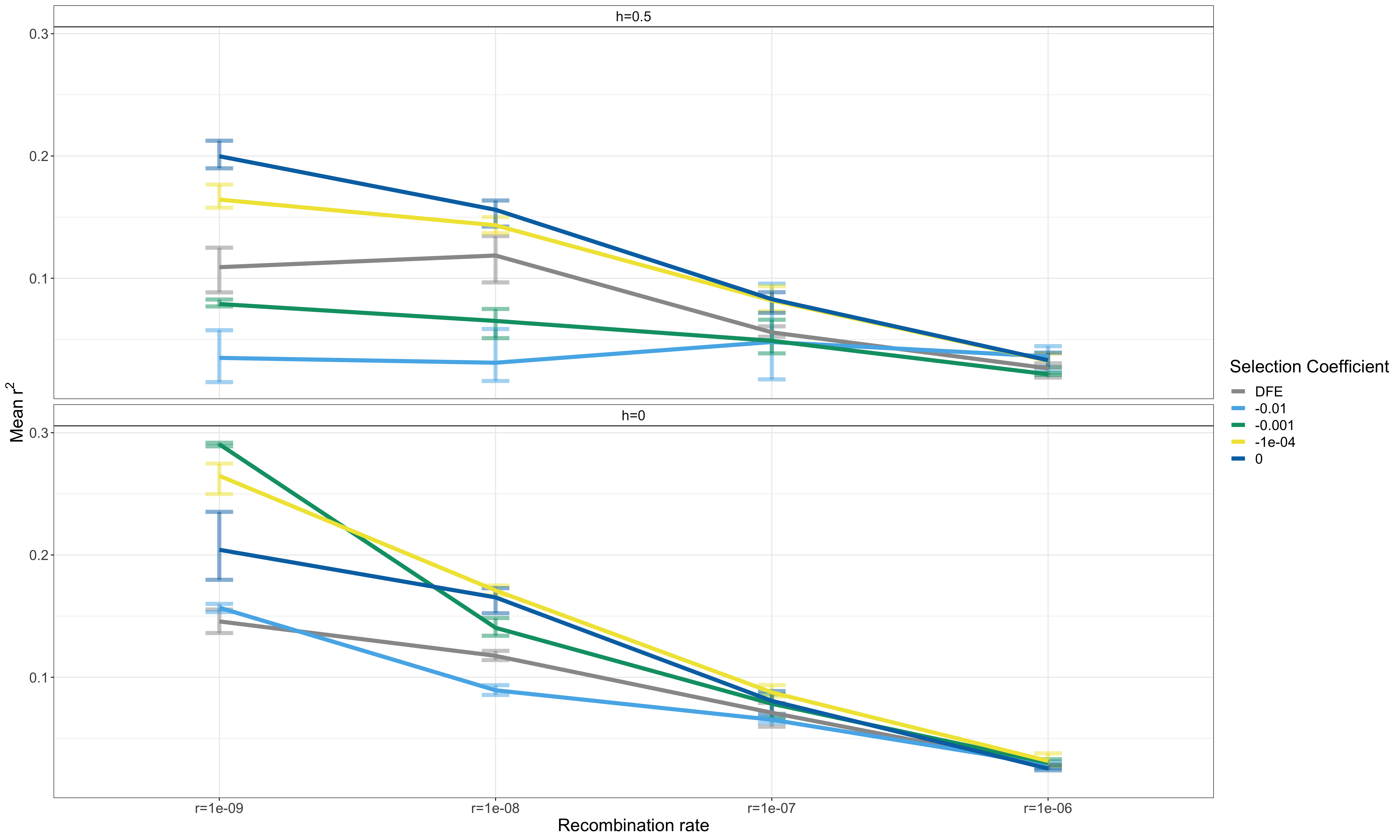

Supplement: S5 Fig — The differences in the decay curves are most apparent for recombination rate r = 1 x 10^−9^ per bp and depend on the dominance coefficient (h) and the selection coefficient (s) of NS mutations. 150 simulation replicates were simulated for each scenario and were split into 3 groups consisting of 50 simulations. For each group the mean value of r2 is shown as the line and the error bars denote the minimum and maximum mean r2 values. (TIFF) [file pgen.1009676.s005.tiff]

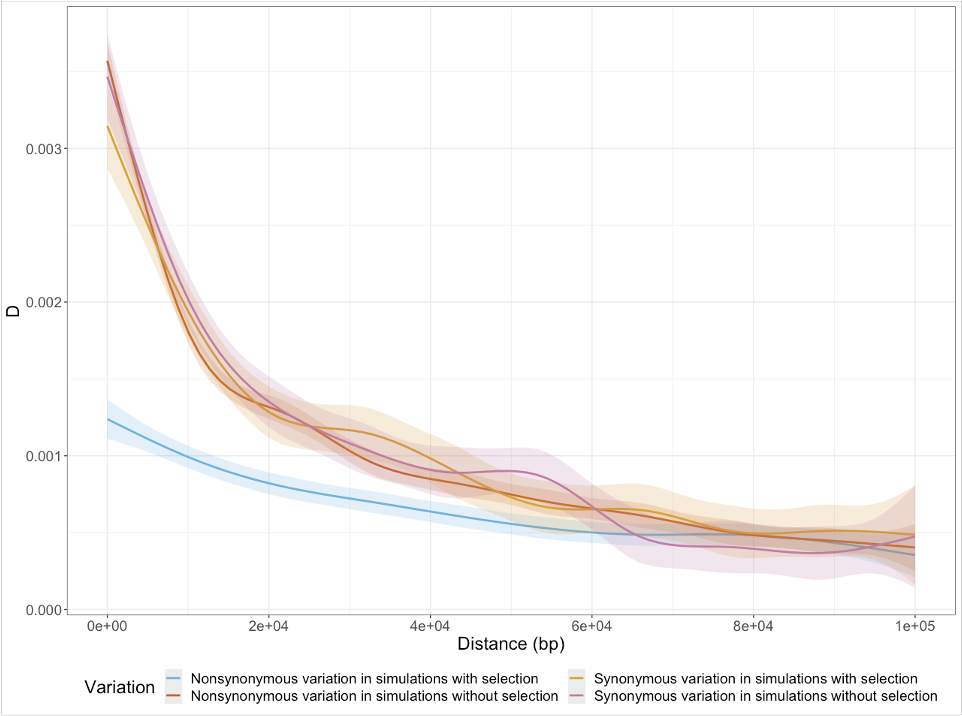

Supplement: S6 Fig — There are two different scenarios shown in the figure: 1) simulations with negative selection, and 2) simulations without negative selection (completely neutral evolution). The blue line is the decay curve of NS doubletons with direct negative selection acting on them. In contrast to the decay curve of the NS doubletons not under negative selection (dark red), the NS doubletons with direct negative selection acting on them (blue) have a more negative D than the other neutral doubletons (S variants in simulations without selection, NS variants in simulations without selection, S variants in simulations with selection). (TIFF) [file pgen.1009676.s006.tiff]

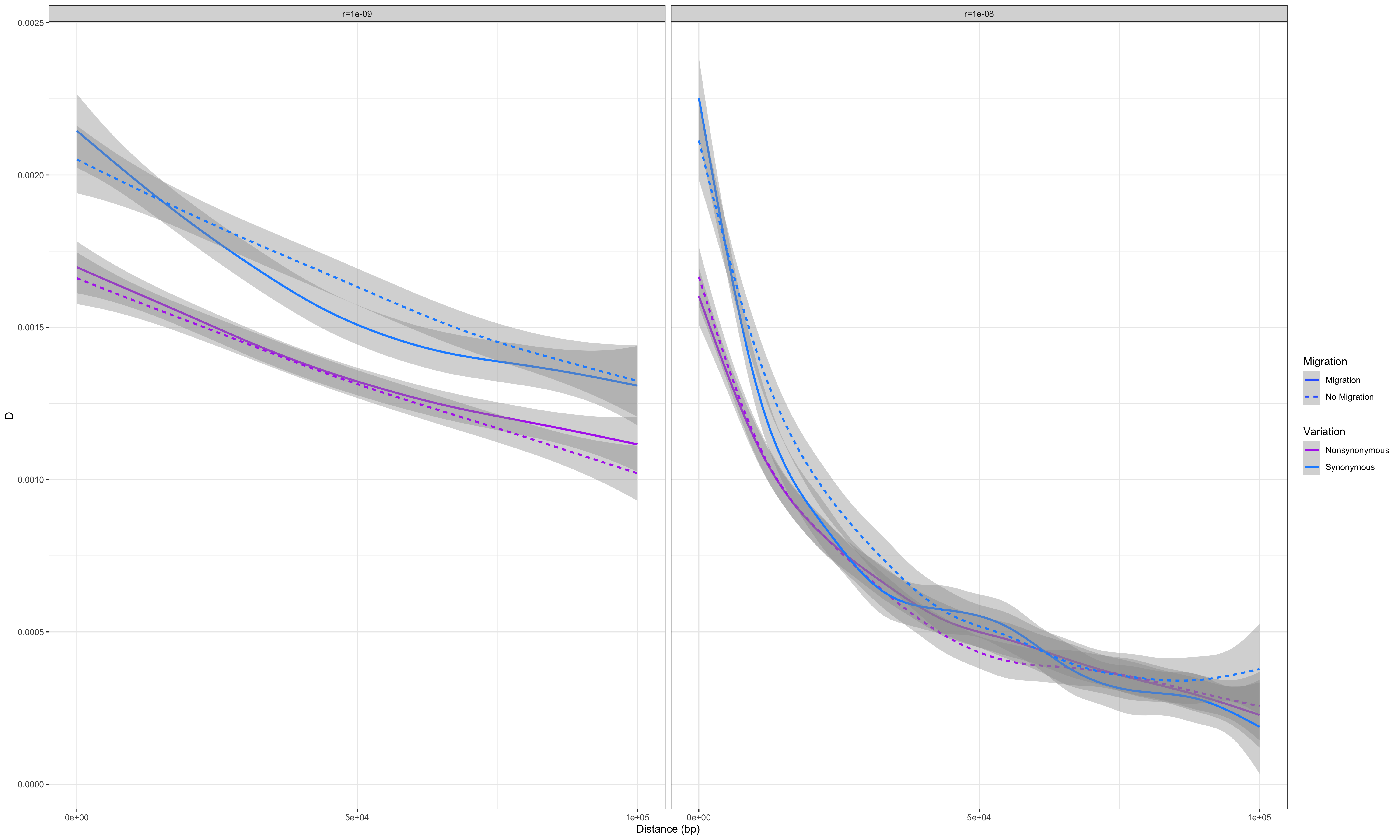

Supplement: S7 Fig — For r = 1 x 10^−8^ per bp, the largest differences between S and NS D is predicted by our simulations to be within the 0–10,000 bp range. In our simulations with r = 1 x 10^−9^ per bp, noticeable differences between S and NS D is predicted by our simulations to be across the 0–100,000 bp range. Additionally, migration appears to qualitatively obscure the difference in D between types of variants at intermediate distances. (TIFF) [file pgen.1009676.s007.tiff]

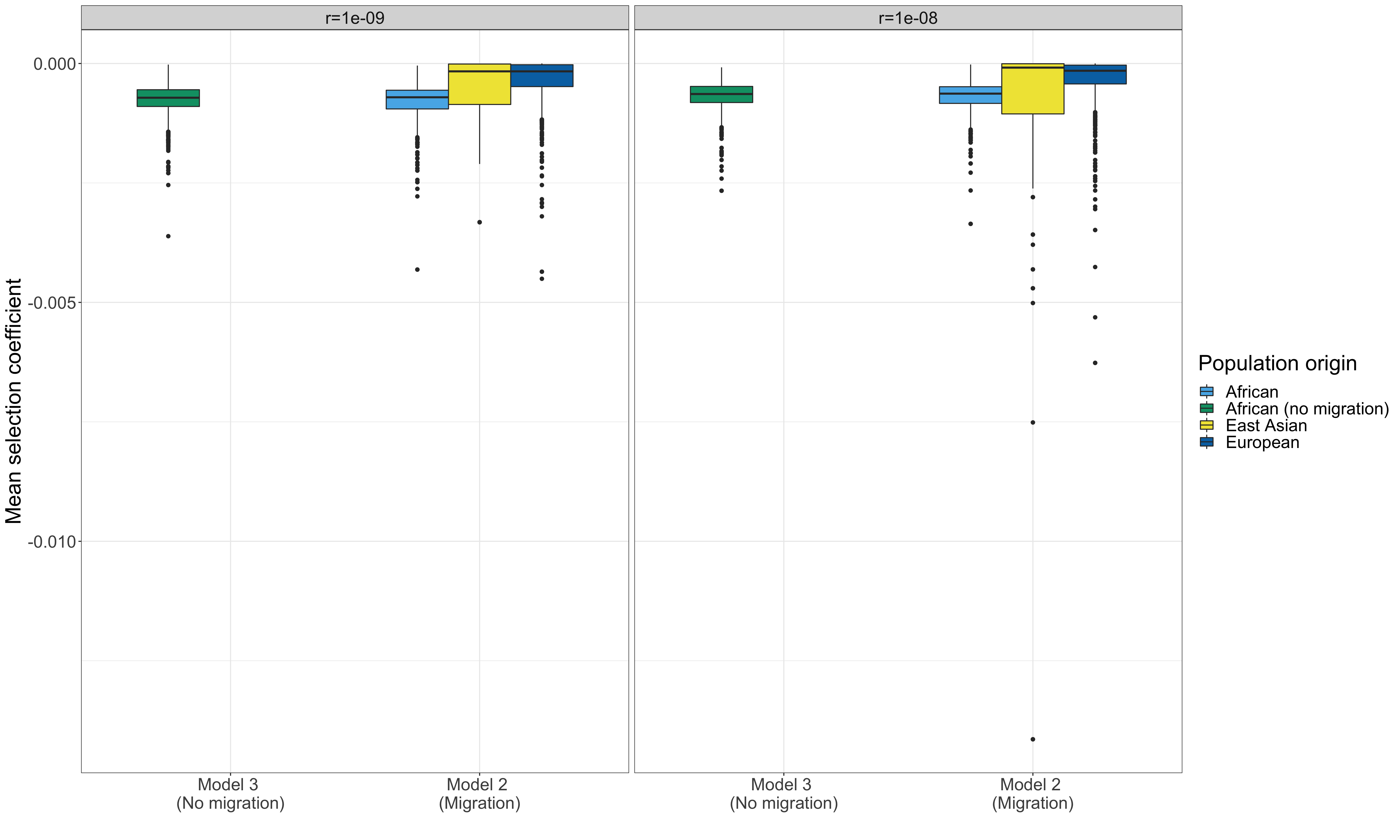

Supplement: S8 Fig — For both r = 1 x 10^−9^ per bp and r = 1 x 10^−8^ per bp, doubletons that appear in our simulated “African” sample, have on average different mean selection coefficients depending on their population of origin. Migration (Model 2) allows for deleterious variants that originated from other populations to appear in our African samples. On average, the doubletons in our sample of African individuals that originate from East Asia and Europe are less deleterious than the doubletons in our sample that originated from Africa. (TIFF) [file pgen.1009676.s008.tiff]

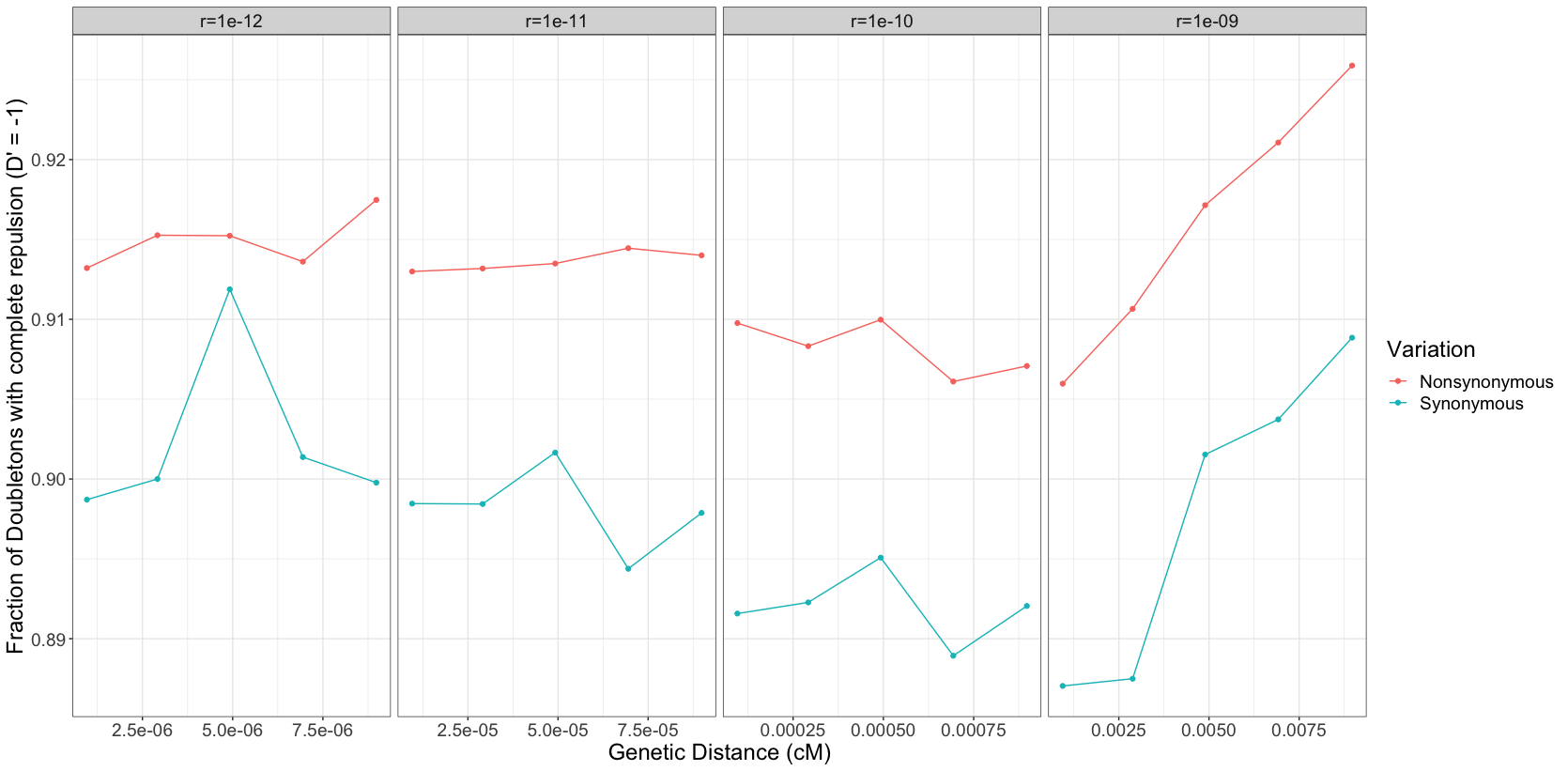

Supplement: S9 Fig — The red line denotes the total fraction of simulated NS doubletons that are in complete repulsion (D’ = -1) and the blue line denotes the total fraction of simulated S doubletons that are in complete repulsion. For each recombination rate, pairwise LD computations were binned into 5 quantiles based on the centimorgan distance between variants. The midpoint between the boundaries of each bin was then computed and defined the bins on the x-axis. (TIFF) [file pgen.1009676.s009.tiff]

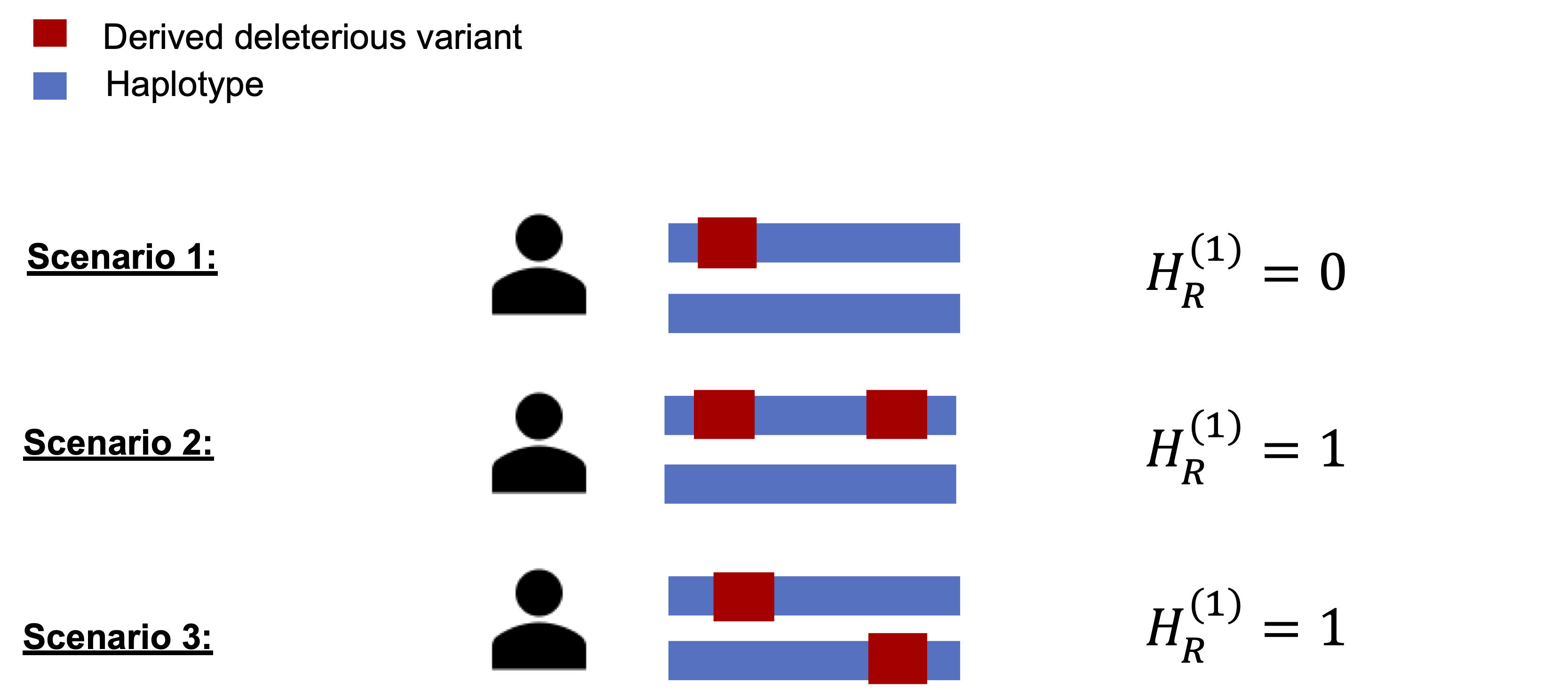

Supplement: S10 Fig — The statistic HR(1) depends on the number of unique pairwise comparisons among singletons within the distance threshold 10 kb (l1 = 1). Additionally, it depends on the number of individuals who are heterozygous at both loci. In both Scenario 2 and 3, the individual is heterozygous at both loci, thus making HR(1) = 1. The statistic nAB depends on the number of haplotypes that contain derived alleles at both loci. In this case, only Scenario 2 contains one haplotype that has both derived variants. HR(1) can also be thought of as an indicator variable taking the value of 1 if an individual is heterozygous at both loci and 0 if not. (TIFF) [file pgen.1009676.s010.tiff]

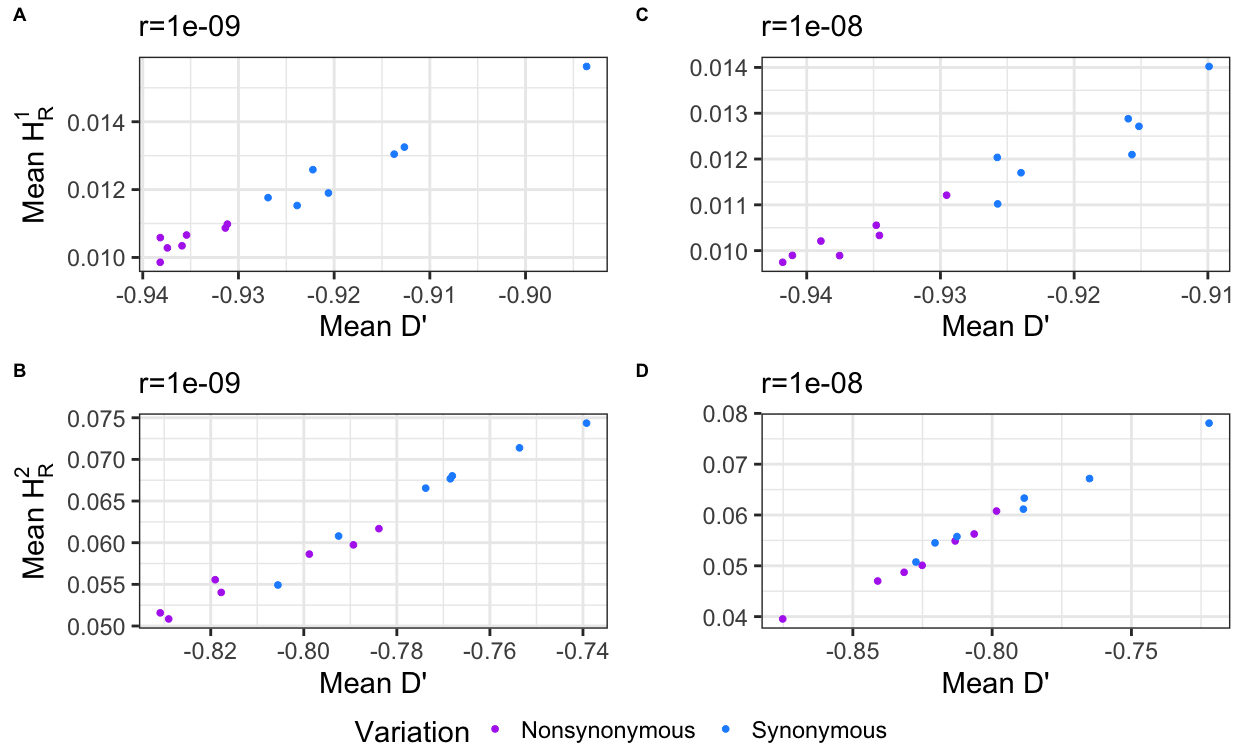

Supplement: S11 Fig — (A) For simulations with r = 1 x 10^−9^ per bp, ⍴ = 0.978 (p-value < 2.2e-16) for the correlation between HR(1) and D’. (B) Also, ⍴ = 0.978 (p-value < 2.2e-16) for the correlation between HR(2) and D’. (C) For simulations with r = 1 x 10^−8^ per bp, ⍴ = 0.947 (p-value < 2.2e-16) for the correlation between HR(1) and D’. (D) Also, ⍴ = 0.996 (p-value < 2.2e-16) for the correlation between HR(2) and D’. The DFE of NS mutations was gamma-distributed with shape parameter 0.186 and expected selection coefficient E[s] = -0.01314833. (TIFF) [file pgen.1009676.s011.tiff]

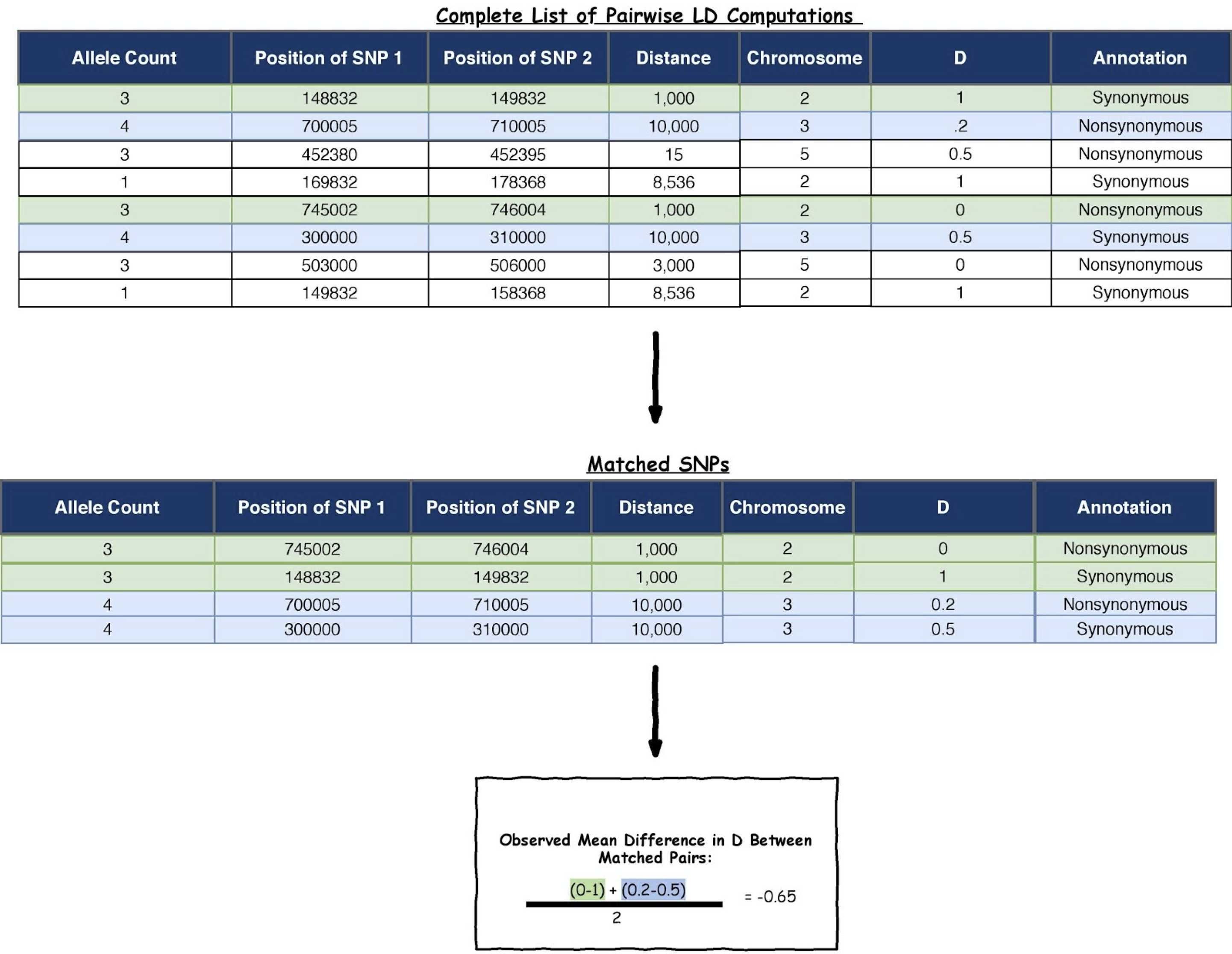

Supplement: S12 Fig — First, sample 50 individuals from a population. Polarize variants and then annotate variants as either NS or S. Second, compute LD summary statistics among pairs of variants that are within 10,000 bp from each other and have the same allele count (AC) and annotation (NS or S). Third, for each pair of NS variants with a computed LD statistic, find one S pair of variants with the same AC, on the same chromosome, and with a similar (<50 bp) distance between variants. Each pair of NS and S pairs constitutes a matched pair. Fourth, compute the mean difference between matched pairs. Fifth, permutate the label (i.e. S or NS) for each pair of SNPs. (TIFF) [file pgen.1009676.s012.tiff]

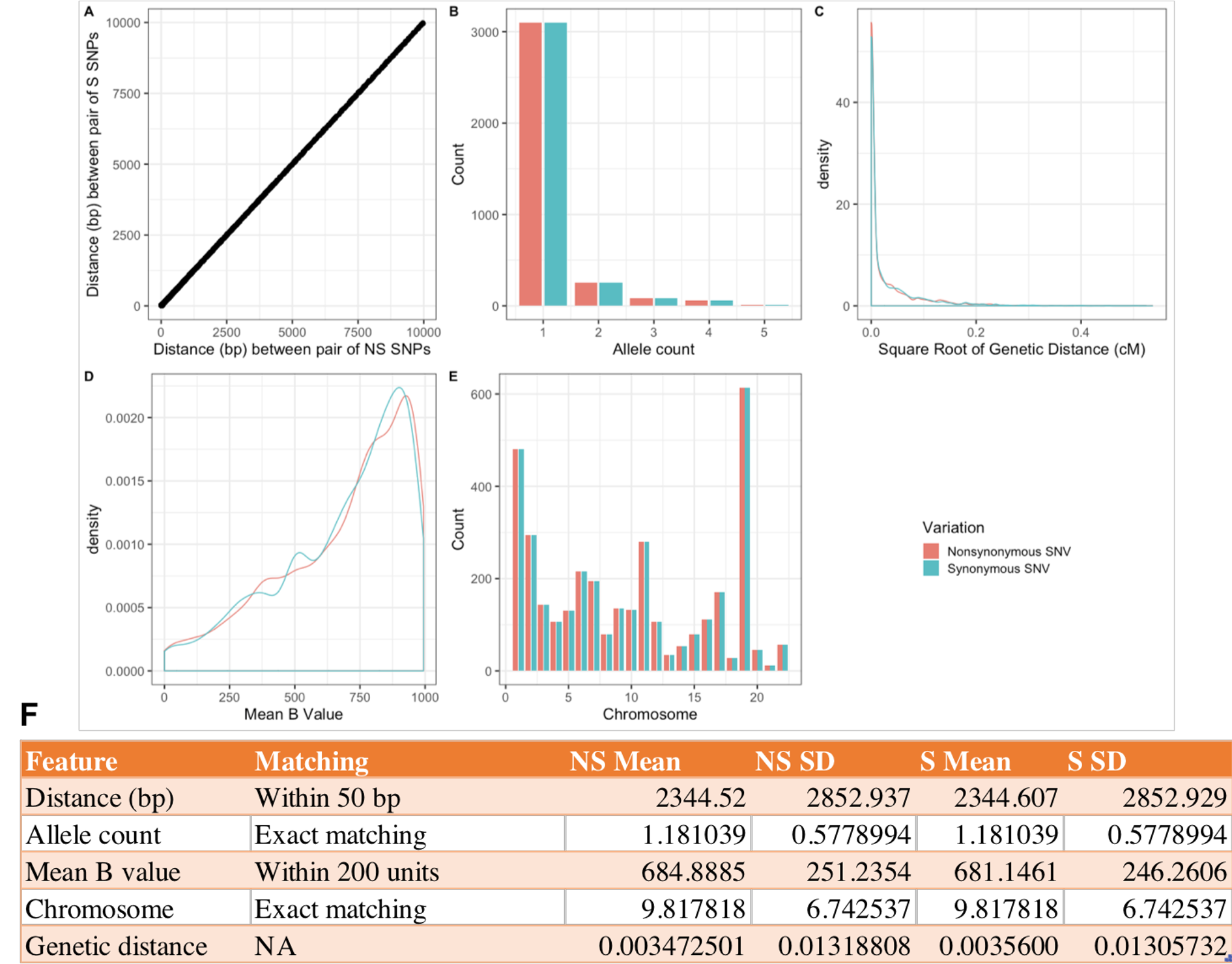

Supplement: S13 Fig — In order to reduce possible confounding effects of different distances, allele counts, levels of background selection, and recombination rate, we implemented a matched pairs scheme. For each pair of NS variants in our data set, we attempted to identify a pair of S variants with similar features. This allowed us to compare pairs of variants with similar distributions and spread of covariates. (A) Physical distance between pairs of S SNPs vs. pairs of NS SNPs after matching. (B) Distribution of allele counts for NS and S SNPs after matching. (C) Distribution of genetic distances between pairs of NS and S SNPs after matching. (D) Distribution of B-values surrounding NS and S SNPs after matching. (E) The number of NS and S SNPs per chromosome after matching. (F) The matching rules and summary statistics of matching success. The “Matching” column denotes the rules we used for matching. “SD” stands for standard deviation. (TIFF) [file pgen.1009676.s013.tiff]

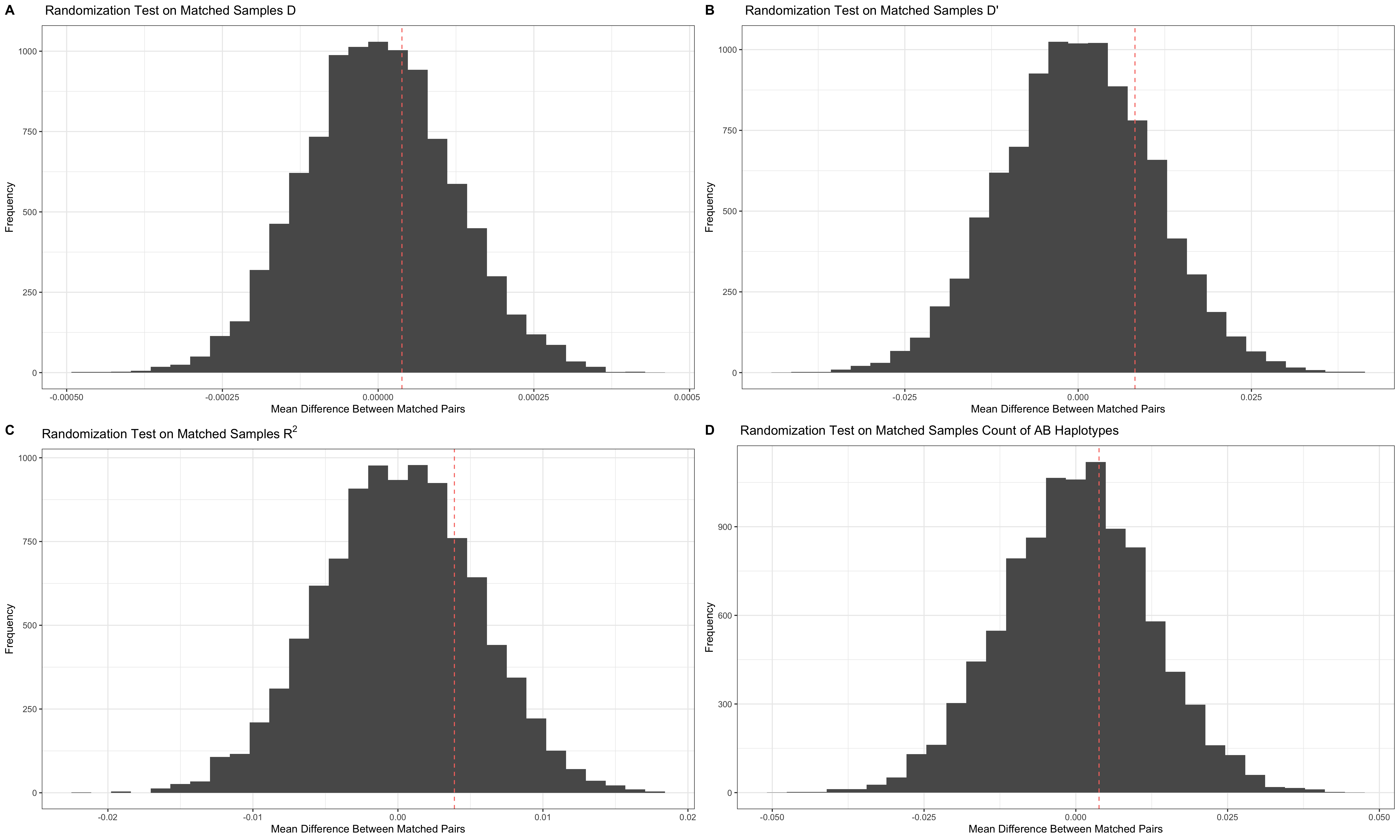

Supplement: S14 Fig — (A) D. (B) D’. (C) r2. (D) Count of the derived haplotype (AB). In simulations without negative selection, none of the LD summary statistics we computed showed a significant difference between NS and S matched pairs. (TIFF) [file pgen.1009676.s014.tiff]

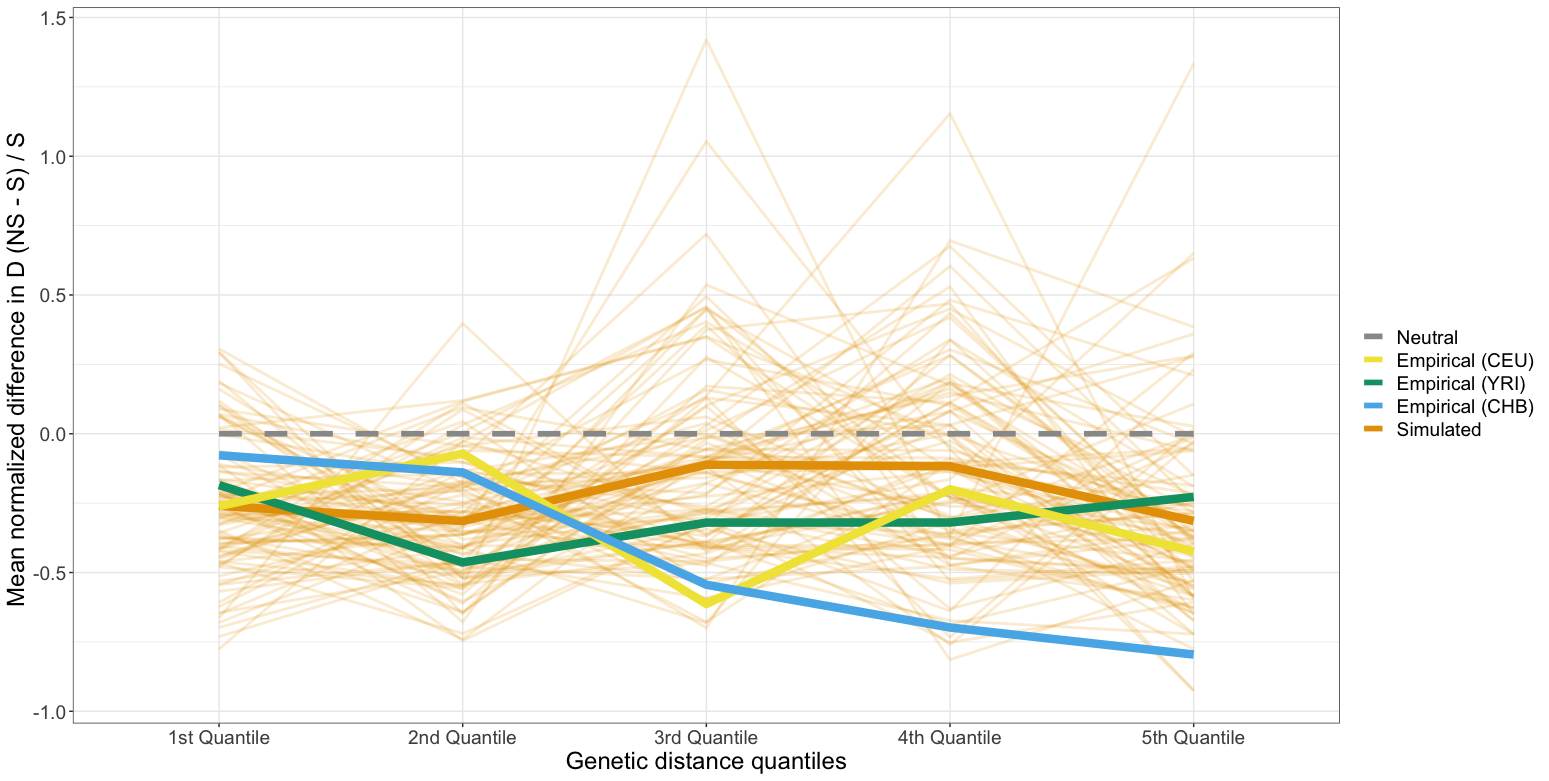

Supplement: S15 Fig — Empirical and simulated data show a deficit in D between NS variants. The lighter orange lines show 100 resamples of the simulated data. Each resample of the simulated data has the same number of variants with the same allele count as the YRI empirical data. All three populations show a more negative D between low frequency NS variants compared to low frequency S variants. (TIFF) [file pgen.1009676.s015.tiff]

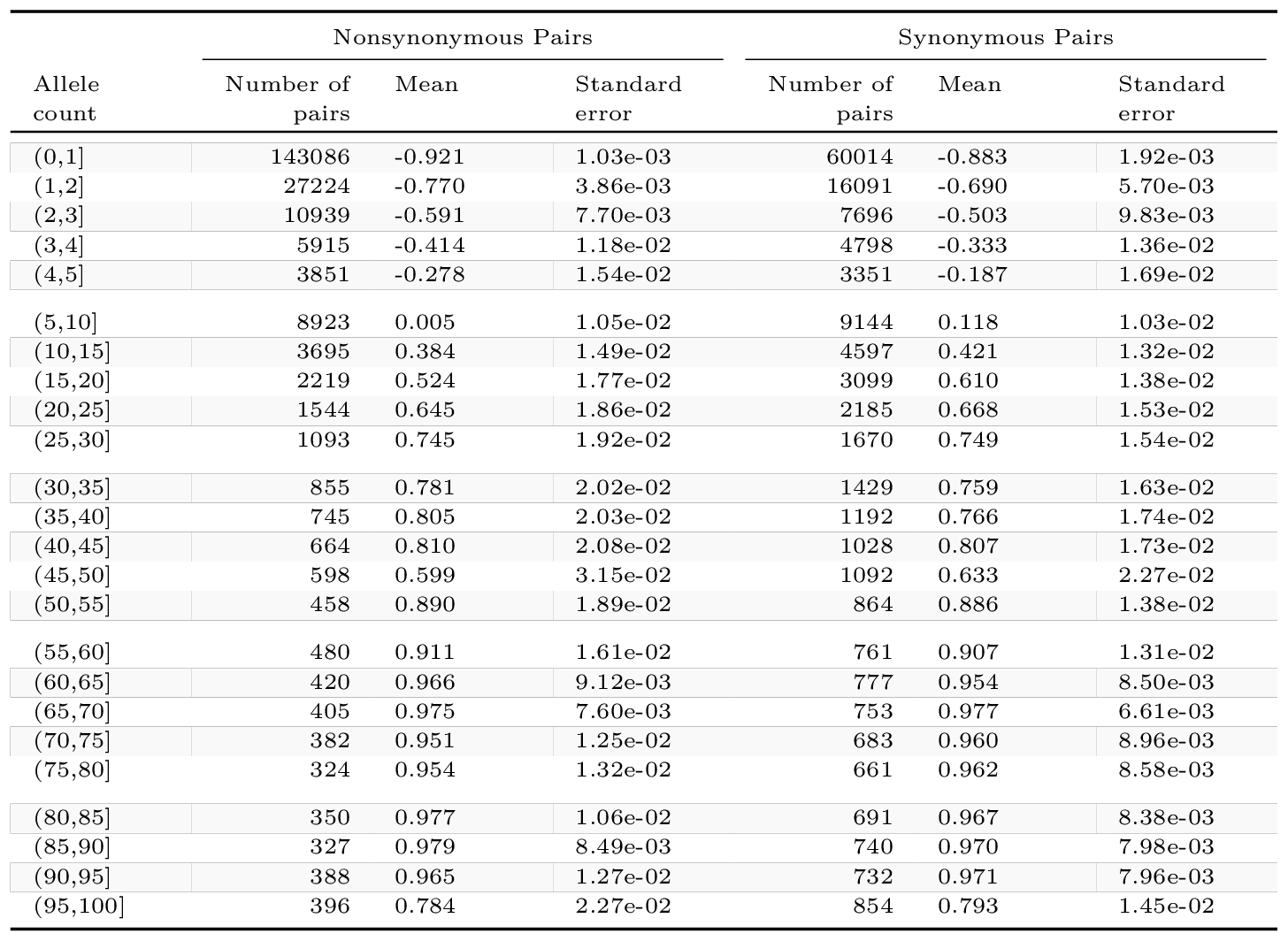

Supplement: S1 Table — In simulations with a gamma distributed DFE, NS pairs of low-frequency variants are predicted to have a less positive D’ compared to S pairs of variants. Generally, as the frequency of variants analyzed increases, D’ becomes more positive and the differences between NS and S pairs decreases. (TIFF) [file pgen.1009676.s016.tiff]

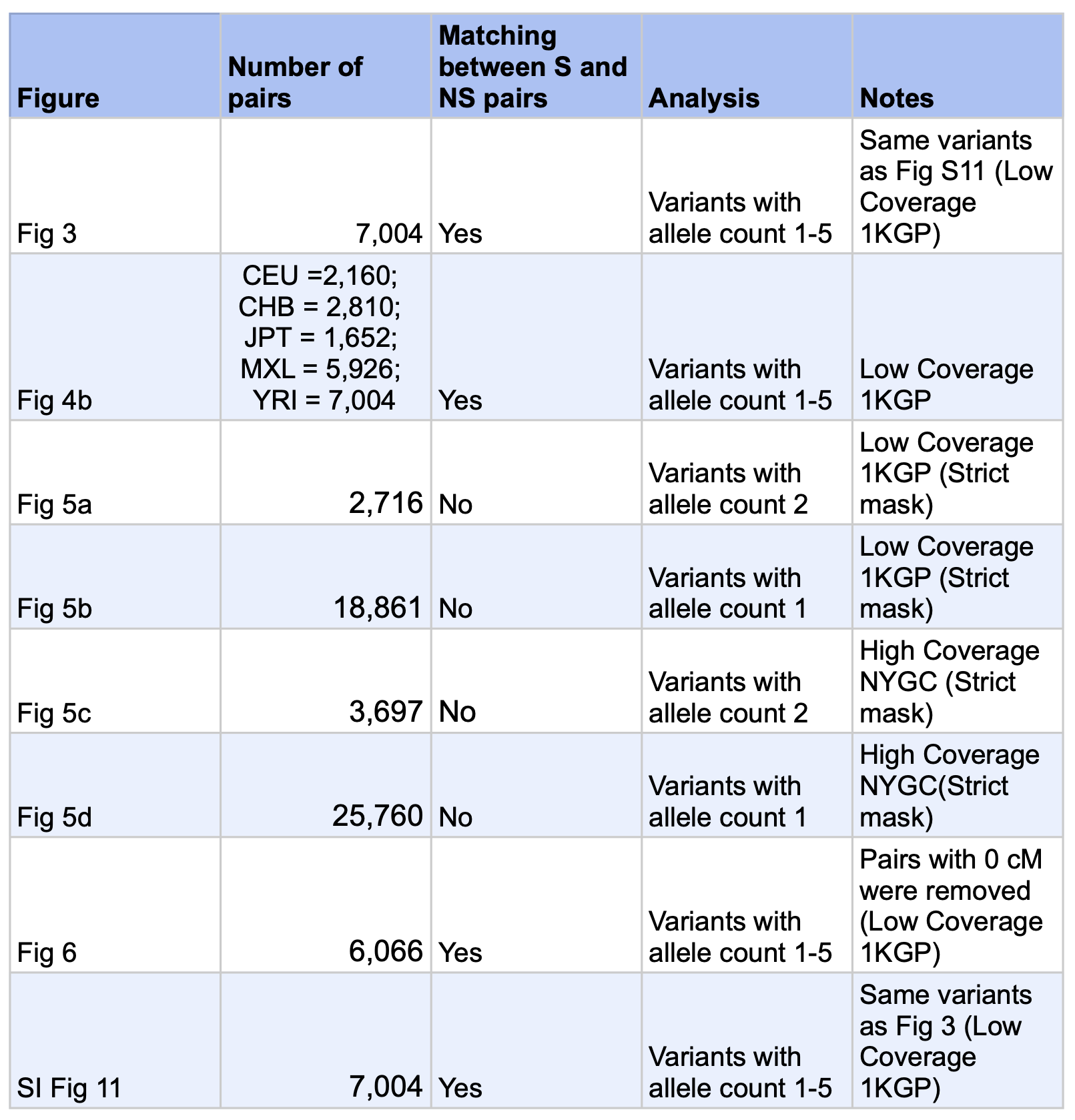

Supplement: S2 Table — The number of pairs of variants in each analysis is in the “Number of pairs” column. Whether or not matching was required between synonymous and nonsynonymous pairs of variants is indicated in “Matching between S and NS pairs” column. The allele count of variants included in the analysis are indicated in the “Allele count of variants” column. The data set from which variants come from and any notes included are indicated in the “Notes” column. If “Matching between S and NS pairs” is “Yes”, then the total number of NS and S pairs are reported in the “Number of pairs” column. (TIFF) [file pgen.1009676.s017.tiff]
