# Supplementary material for: Negative linkage disequilibrium between amino acid changing variants reveals interference among deleterious mutations in the human genome
Source: PLoS Genet. 2021 Jul 28;17(7):e1009676. doi: 10.1371/journal.pgen.1009676 (PMC8351996; doi:10.1371/journal.pgen.1009676)
Supplement: S1 Text — (PDF) [file pgen.1009676.s018.pdf]

# S1 Text

### Predicted effect of negative selection on linkage disequilibrium decay

To gain an overall understanding of how negative selection can impact LD patterns, as measured by the squared correlation coefficient between SNPs ($r^2$), we first simulated populations under demographic Model 1 (constant-size population) where NS mutations all have the same effects on fitness (**S2 Fig**). In **S2 Fig**, we show results for all variants, regardless of frequency or functional annotation. The strength of negative selection, degree of dominance, and recombination rate, all interact and affect the mean $r^2$ decay with physical distance between SNPs (**S2 Fig**). This effect is most visible in simulation replicates with low recombination rates. Specifically, under the additive model, i.e., where $h$ = 0.5, simulations with weakly to moderately deleterious ($-0.001<s<-0.01$) mutations have lower values of $r^2$ than do simulations with only neutral variants. However, simulations with deleterious variants with $s$= -0.1 tended to have the highest mean $r^2$ for a given physical distance, mirroring the LD patterns of neutral variants. This is likely in part due to the fact that strongly deleterious mutations are not expected to segregate within a population for long. Therefore, the effects of interference are diminished [1]. However, it is still possible to observe the consequences of background selection from these deleterious mutations on overall patterns of LD. Zeng and Charlesworth have previously shown that LD can be elevated by background selection to an extent that is well summarized by a change in effective population size [2]. This result is concordant with our simulations here.

We next explored whether the dependence of LD on distance and recombination rate was solely mediated by the genetic map distance separating the variants. First, in entirely neutral simulations, nonsynonymous variants simulated with $r$=1 x $10^{-9}$ at 10kb, the mean $r^2$ is approximately 0.145. At 1kb simulations with $r$=1 x $10^{-8}$ the mean $r^2$ is approximately 0.15. Thus, for neutral simulations, the decay in LD appears to be entirely due to the genetic distance between variants, as expected. However, the pattern is more complex in simulations with selection. For example, when deleterious variants arise with a selection coefficient of -0.0001, at 10kb the mean $r^2$ is approximately 0.08 when $r$=1 x $10^{-9}$. However, at 1kb and $r$=1 x $10^{-8}$, the mean $r^2$ is approximately 0.125. If the LD decay was fully mediated by the map distance, the mean $r^2$ also should be around 0.08. This difference is due to the effects of interference selection coming from other nearby linked variants affecting the behavior of the pair of variants in question.

For simulations with recessive deleterious mutations ($h$=0), there is a complex relationship between the selection coefficient and LD decay (**S2 Fig**). Here moderately and strongly deleterious ($s$=-0.01 and -0.1) variants have the lowest values of $r^2$ at a given physical distance. However, simulations with NS variants with selection coefficients of -0.0001 have the largest mean $r^2$. Thus, for the recessive case, there is no consistent ordering of how selection coefficients affect genome-wide $r^2$ decay with physical distance.

For simulated genomes with recombination rates greater than 1 x $10^{-9}$ per bp, the effect of negative selection on mean $r^2$ and its decay is less pronounced. Here, simulations with

deleterious variants that arise with drastically different selection coefficients displayed similar $r^2$ decay patterns. Furthermore, as the recombination rate increased, the overall magnitude of $r^2$ decreased, as expected given previous work [3,4].

**Additional References**


1. Comeron JM, Williford A, Kliman RM. The Hill-Robertson effect: evolutionary consequences of weak selection and linkage in finite populations. Heredity. 2008;100: 19–31. doi:10.1038/sj.hdy.6801059
2. Zeng K, Charlesworth B. The joint effects of background selection and genetic recombination on local gene genealogies. Genetics. 2011;189: 251–266. doi:10.1534/genetics.111.130575
3. Hill WG, Robertson A. Linkage disequilibrium in finite populations. Theor Appl Genet. 1968;38: 226–231. doi:10.1007/BF01245622
4. Pritchard JK, Przeworski M. Linkage disequilibrium in humans: models and data. Am J Hum Genet. 2001;69: 1–14. doi:10.1086/321275
