# Supplementary material for: Negative linkage disequilibrium between amino acid changing variants reveals interference among deleterious mutations in the human genome
Source: PLoS Genet. 2021 Jul 28;17(7):e1009676. doi: 10.1371/journal.pgen.1009676 (PMC8351996; doi:10.1371/journal.pgen.1009676)
Supplement: S2 Text — (PDF) [file pgen.1009676.s019.pdf]

S2 Text

Effect of negative selection and complex demography on LD

We examined how complex, non-equilibrium demography impacts LD between deleterious variants. We hypothesized that although population growth and migration all perturb levels of LD, we should still be able to detect interference by comparing LD between pairs of deleterious NS variants to the LD between pairs of neutral S variants. To test this, we simulated under Model 2, which is a more realistic model for human demography, containing exponential population growth, and migration (**S1 Fig**). These simulations also use a distribution of fitness effects for deleterious mutations (see Materials and Methods). Here we still see the effects of interference generating an excess of negative LD among derived deleterious doubletons as NS doubletons have lower values of *D* than do neutral S doubletons (**S7 Fig**). Again, the difference between functional annotations (NS or S) is greater for SNPs at low recombination rates. These findings suggest that the relative excess of negative LD can be detected in human-like demography and is not limited to populations with a constant long-term N_e and in equilibrium.

We hypothesized that migration across populations might disrupt the strength of the correlation between allele frequency and fitness effects, which in turn could affect the strength of interference and LD. In Model 2 (which includes migration between populations), low frequency variants in a sample might be at low frequency due to having just recently arrived in the population via migration. These low-frequency variants could be at higher frequencies in other populations giving them a high probability of migrating to a new population. As such,

these doubletons may be at low frequency due to the fact that they have recently arrived to a new population, rather than being actively kept at low frequency by negative selection. To test for this effect, we looked at the relationship between the mean selection coefficient of doubletons based on their population of origin (**S8 Fig**). Although we sample from the African population, there are variants in the sample that arose in other populations. On average, variants that originated from different populations then migrated into Africa are less deleterious (**S8 Fig**). Additionally, we hypothesized that, if migration is the source of less striking differences in LD between NS and S variants in Model 2, then the same demographic model without migration across populations will show a larger difference in LD patterns between neutral and deleterious SNPs. To test this hypothesis, we conducted additional simulations under the same demographic history as Model 2, though without migration (Model 3). Model 3 shows a slightly greater excess of negative LD among derived NS variants compared to S variants (**S7 Fig** dashed lines) than that seen under Model 2 (**S7 Fig** solid lines). However, because this pattern is quite subtle, it suggests that migration has had a limited impact on differences in patterns of LD across different types of SNPs.

Additionally, in our Model 3 simulations, we quantified differences in the proportion of pairs of NS and S doubletons in complete repulsion (i.e. D' = - 1; **S9 Fig)**. We hypothesized that the more negative LD statistics of simulated deleterious NS variants is due to more pairs of NS variants being in complete repulsion (D' = - 1) than were pairs of S variants. In our study, pairs of doubletons in complete repulsion refers to the case where a pair of doubletons are distributed across haplotypes in a way such that derived variants never co-occur on the same haplotype in

our sample. Simulations under Model 2 predict NS pairs of doubletons should be more often in complete repulsion (*D′* = -1) than S pairs of doubletons (**S9 Fig**). In sum, simulations with negative selection predict deleterious doubletons should have lower values of mean *D′* relative to neutral doubletons.
