# Supplementary material for: Negative linkage disequilibrium between amino acid changing variants reveals interference among deleterious mutations in the human genome
Source: PLoS Genet. 2021 Jul 28;17(7):e1009676. doi: 10.1371/journal.pgen.1009676 (PMC8351996; doi:10.1371/journal.pgen.1009676)
Supplement: Attachment — Submitted filename: Response to Reviewers.R1.final2.pdf [file pgen.1009676.s020.pdf]

Response to Reviewers

Reviewers' comments are in Black: Authors' responses are in Blue

Note that all line numbers refer to the clean copy.

Major presentation comment

The structure of the Results section is difficult to follow. Right now, there are many rounds of simulations, comprising more than half the methods section, but after these many subsections there is a one-sentence statement that none of the LD metrics computed in simulations can actually be computed on real data due to lack of phased data (I disagree with this statement; see minor comment 7 below). Then, two new methods are introduced (H_R(j) and a permutation test), and it's not clear what the relationship is between them (I assume the permutation test is applied to H_R(j), but this isn't stated). Then, results on real genotypes are presented, and at the same time, new simulations are performed to show that the new methods work.

I suggest the following organization for the results section: (0) optionally, describe a small set of motivating simulations that illustrate the effect that you wish to quantify;

(1) describe new methods and why they are needed, including H_R(j) and the permutation test;

(2) perform simulations, and devote at least one simulation subsection to demonstrating that the new method/metric performs as intended;

(3) apply new method/metric to real genotypes.

We thank the reviewer for suggesting that we restructure the presentation of the Results section. In the revised manuscript, we have greatly reduced the length of text describing simulation results and now only present one main text figure (**Fig1**) showing the general results from the simulations with traditional LD statistics. We have moved some of the results using other LD statistics and other sets of variants into **S1 Text**. We have also moved some of the text presenting simulation results from alternate demographic models to **S2 Text**.

Beyond shortening the text describing the simulation results, we also have changed the order of the Results section based on the reviewer's suggestions. Specifically, we first describe the predicted effect of negative selection on linkage disequilibrium decay. Then we describe our new H_R^j statistic, and then the matched-pairs permutation test. Lastly, we apply the new method to real genotype data from humans.

1. Figure 1: Is the dependence of LD on bp distance and recombination rate fully mediated by the map distance? I.e. is LD approximately the same at distance=1kb and rate=10-7 vs d=10kb and r=10^-8? If not, please explain why. If so, consider changing the x axis to map distance and reducing the number of figure panels from 8 to 2. Also, please set the minimum y-axis value to zero on all panels.

As suggested by the reviewer, we have moved some of the simulation results to SI material. Thus, the main text Figure 1 from the initial version of the manuscript to which the reviewer is referring to here is now **S2 Fig** in the revised manuscript.

The dependence of LD on distance and recombination rate does not appear to be solely mediated by the map distance. We examined this by looking at the LD at distance 1kb vs the LD at distance 10kb of our different simulation replicates. When our simulations have nonsynonymous variants that arise with s=0 (neutral), at 10kb, the mean r^2 is approximately 0.145. At 1kb simulations with r=1e-08 the mean r^2 is approximately 0.15. This is the expected pattern if the dependence of LD decay on distance was mostly mediated by the map distance. However, there are differences in simulations with selection. For example, when deleterious variants arise with a selection coefficient of -0.0001, at 10kb the mean r^2 is approximately 0.08 when r=1e-09, but at 1kb and r=1e-08 the mean r^2 is approximately 0.125. If the LD decay was fully mediated by the map distance at r=1e-08, the r^2 should be around 0.08. This difference is due to the effects of interference selection coming from other nearby linked variants affecting the behavior of the pair of variants in question.

We now have set the minimum y-axis value to zero on all panels. Additionally, we reduced the number of figure panels from 8 to 4. The new **S2 Fig** is shown below:

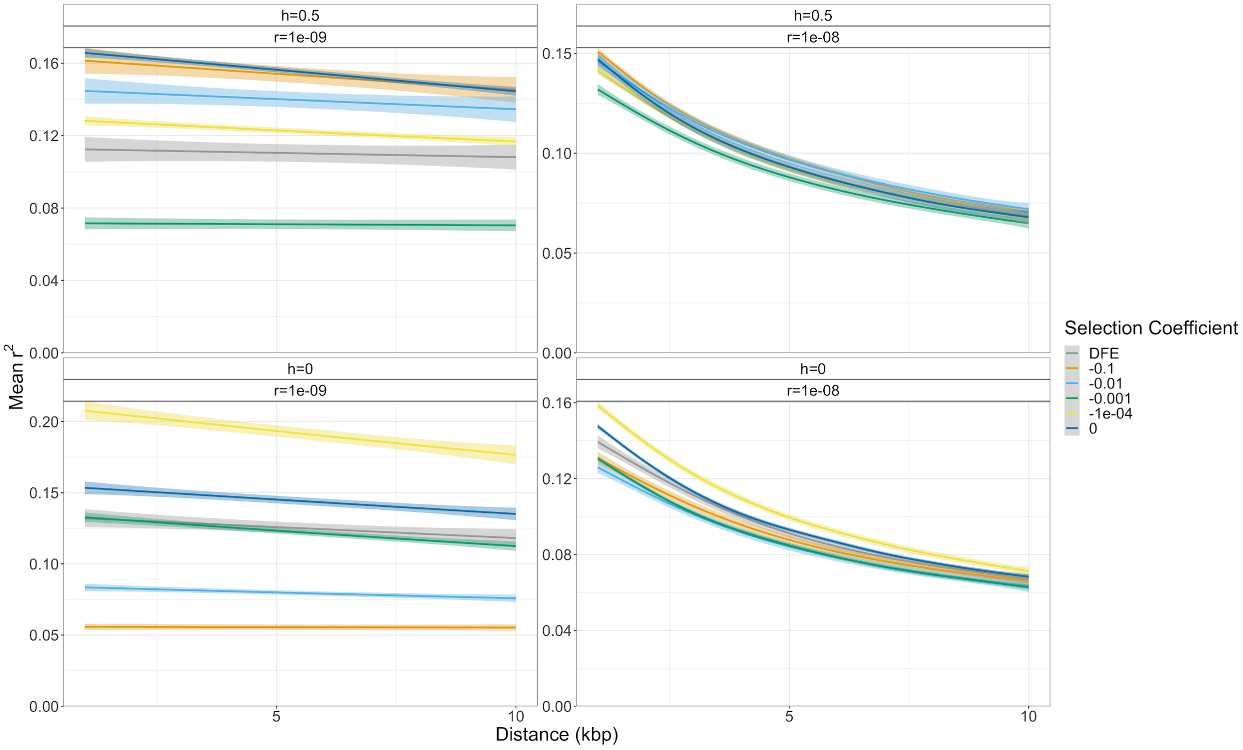

We also now discuss this issue of the LD decay not depending solely on map distance between variants in **S1 Text** of the revised manuscript. Specifically, we write, “We next explored whether the dependence

of LD on distance and recombination rate was solely mediated by the genetic map distance separating the variants. First, in entirely neutral simulations, nonsynonymous variants simulated with r=1e-9 at 10kb, the mean r^2 is approximately 0.145. At 1kb simulations with r=1e-08 the mean r^2 is approximately 0.15. Thus, for neutral simulations, the decay in LD appears to be entirely due to the genetic distance between variants, as expected. However, the pattern is more complex in simulations with selection. For example, when deleterious variants arise with a selection coefficient of -0.0001, at 10kb the mean r^2 is approximately 0.08 when r=1e-09. However, at 1kb and r=1e-08, the mean r^2 is approximately 0.125. If the LD decay was fully mediated by the map distance, the mean r^2 also should be around 0.08. This difference is due to the effects of interference selection coming from other nearby linked variants affecting the behavior of the pair of variants in question."

2. "no consistent ordering of how selection coefficients affect genome-wide r2 decay with physical distance": what is the intuition for why this occurs?

Thank you for this question. We agree that it is important to explore this aspect in greater detail. We believe that this effect is due to the complex interplay of HRI effects combined with a heterosis effect (i.e. associative overdominance) whereby recessive deleterious mutations are masked from selection in the heterozygous state. If the recombination rate is low, then haplotypes carrying multiple deleterious mutations may increase in frequency because they can be efficiently masked together in the heterozygous state if they're all on one haplotype.

Revised Text

(lines 246-249) The behavior of recessive deleterious mutations is due to the complex interplay of HRI effects combined with a heterosis effect (associative overdominance) whereby recessive deleterious mutations are masked from selection in the heterozygous state [46]. This effect may be exaggerated if there is little recombination and the deleterious alleles arise on the same haplotype, leading to positive LD.

3. "Human-like exomes… human-like levels of negative selection": when model parameters are chosen to match human-inferred values, do resulting observable quantities actually match what we observe in humans? Is the allele frequency spectrum similar? Is the average LD similar?

Our comment about "human-like exomes" was referring to the length distribution of human exons. Additionally, "human-like" levels of negative selection refers to the distribution of fitness effects (DFE) that we used which was inferred from nonsynonymous variants in humans (see Kim et al. 2017). We added additional clarification to the main text of the revised manuscript (see below). Our chosen parameters should match the allele frequency spectrum from human data as these parameters were inferred from human data.

We did not incorporate empirically measured recombination maps into our simulations, and simulated under a constant recombination rate. Thus, the average LD is from our simulations is not expected to

exactly match that seen in the empirical data. We now talk about this limitation of our study in the revised manuscript and highlight that these model parameters are not chosen to match empirical data.

Revised Text

(lines 467-477) While we use simulations to quantify the effects of Interference on LD statistics, a limitation of this study is that we do not simulate full human genomes and directly assess whether the model parameters can fully explain the empirical data. The shape and scale of the DFE that we simulated under closely recapitulates empirically observed site frequency spectrum in human genetic variation data [40]. However, for tractability, we did not incorporate empirically measured recombination maps into our simulations, and instead simulated under uniform recombination rates. Because of this, the LD decay curves in our sample may not quantitatively match the curves seen in empirical data. With these limitations in mind, we compared trends from these simulations qualitatively to our empirical data. Future work can test whether realistic genetic maps, functional annotations, and a DFE can match genome-wide patterns of LD.

4. "As the degree of LD between a pair of doubletons also is influenced by the amount of recombination that occurs between them, it is possible that there could be different genetic distances (cM) between pairs of NS and S SNPs": I don't understand this sentence. Should it instead state that different genetic distances between NS vs S SNP pairs could be an alternate explanation for differences in LD?

Thank you for bringing this to our attention. We have rephrased this sentence to be clearer.

Revised Text
(lines 259-262) As the degree of LD between a pair of doubletons also is influenced by the amount of recombination that occurs between them, we wanted to ensure that the results shown above were not driven by different amounts of recombination occurring between pairs of NS SNPs and pairs of S SNPs.

5. Effect of background selection on LD between S variants: this is interesting, and I think it merits further investigation, but Figure S5 does not convince me that the effect is real. Is the slight difference that is observed at d<500bp statistically significant? Is it explained by differences in allele frequency spectrum? What is the selection coefficient for NS variants in these simulations, and might the effect be stronger under weak selection than under strong selection?

We agree that the effects presented in **S5 Fig** of the original version of the manuscript may not have been fully convincing. In the interest of making the paper more focused, as suggested by the comments from the reviewers, we decided to remove the discussion about the effect of background selection on LD between S variants. This is a topic that could merit its own further investigation in future work.

6. Proportion of S vs NS variants with D=-1: this long paragraph doesn't cite any main or supplementary display items, and it doesn't seem especially well motivated – given that E(D) is already shown to be different, is there any scenario where P(D=-1) would not be different? Given that P(D=-1) is not quantified in analyses of real data, I think that this subsection could be deleted.

Thank you for pointing this out. We agree and have removed this section from the main text of the manuscript. We now include a much briefer discussion about the proportion of doubleton SNP pairs in complete coupling (*D'*=1) or repulsion (*D'*=-1) in **S2 Text** and **S9 Fig** as we believe that the proportion of pairs in coupling/repulsion may provide more intuition for the effects rather than just focusing on the average value.

7. "traditional LD statistics… require phased haplotype information": It's not clear to me that this is true. Let r_phased be the correlation between the haploid genotypes for two markers, and r_diploid be the correlation between the diploid allele counts. Under Hardy-Weinberg, r_phased=r_diploid. Are potential HWE deviations the reason for needing something more sophisticated? Is observable deviation from HWE even possible for doubletons? Overall, I feel that H_R(j) could be motivated more clearly. (However, this manuscript does not need a nontrivial new statistic to be a valuable contribution).

The LD statistics D, r^2, and D' when used on a sample from a finite population require some form of phased haplotype information. The reviewer is correct in that under Hardy Weinberg Equilibrium (HWE), the correlation between the diploid allele counts (as denoted by {0,1, or 2}) is the correlation between the haploid genotypes for two markers. However, when studying sites influenced directly by natural selection, the assumptions of HWE are violated. Further, the performance of computational methods to impute/phase variants become less accurate for rare variants [47,48], which are the focus of our study. Our $H_R^{(j)}$ statistic is similar to r^2 but there are a few differences. $H_R^{(j)}$ is focused on pairwise comparisons of variants of specific frequencies (doubletons only, or singletons only) while genotype r^2 is not. Additionally, genotype r^2 is based on covariances and variances while $H_R^{(j)}$ is focused on counts.

While it is unclear whether violations from HWE will impact inferences made on real data, given that it is possible to use other statistics to capture associations between diploid genotypes that are more biological interpretable, such approaches are justified. We now better clarify this point in the revised manuscript.

Revised Text

(lines 284-296) The standard LD statistics r^2, D, and D' are based on counts of the number of haplotypes that contain both derived alleles at both loci (p_{AB}), which require phased haplotype information. Often when studying nonmodel organisms or unprocessed genotype data we typically do not have this information and would have to rely on computational phasing. The performance of computational methods to impute or phase variants becomes more challenging for rare variants [47,48], which are the focus of our study. Further, when studying sites influenced directly by natural selection, the assumption of HWE may be violated and the correlation between the diploid allele counts (as denoted by {0,1,or 2}) may not equal the correlation between the haplotypes for two markers.

Here we introduce new statistics that can be directly computed from diploid genotype data to quantify whether derived alleles are more likely to be coupled (i.e. on the same haplotype) or in repulsion (i.e. on different haplotypes) for different functional annotations.

8. D′ values of S vs NS variants: are the observed values, including the apparently non-monotonic trend, consistent with simulations? Are the S values consistent with neutrality?

The overall pattern of lower values of *D′* between pairs of NS variants compared to pairs of S variants is consistent with simulations. This observation (which **Fig 3** shows in empirical data) is mirrored by figures such as **S1 Table** and **Fig 4A**. **S1 Table** shows the mean *D′* of matched pairs of simulated NS and S SNPs across allele counts in a sample of *n*=50 diploid individuals. On average, a more negative *D*′ is seen across pairs of low frequency NS polymorphisms. The non-monotonic trend we observe in the empirical data appears to be sensitive to things like dominance coefficients distribution of fitness effects, and recombination rates.

Some of the non-monotonic trends may also be due to the limited number of SNP pairs in particular recombination rate bins leading to imprecise measurements of the mean *D′*. Indeed **Fig 6** of the revised manuscript shows how downsampling the simulation replicates to have the same number of SNP pairs as do the empirical data (3,160 pairs of NS SNPs and 2,906 pairs of S SNPs) shows fairly high variance, consistent with the empirical observations.

We now clarify this point in the revised manuscript where we write, (lines 419-424) "Some of the non-monotonic trends observed in our analysis of empirical data may also be due to the limited number of SNP pairs in particular recombination rate bins leading to imprecise measurements of the mean *D*. **Fig 6** shows how downsampling the simulation replicates to have the same number of SNP pairs as do the empirical data (3,160 pairs of NS SNPs and 2,906 pairs of S SNPs) induces fairly high variance, consistent with the empirical observations."

9. Figure 4: I suggest to calculate standard errors and include error bars for panel A; to use a linear x-axis scale in panel A with round numbers as x-tick values; to reduce the number of subpanels in B from 3 to 1, as all three subpanels convey the exact same information; and to move panel C content to simulations section.

We thank the reviewer for these suggestions, which we have implemented. First, we have reduced the number of panels in **Fig 3** of the revised manuscript (**Fig 4** of the previous version of the manuscript). The other panels have been moved to **S14 Fig**. We also now present error bars on **Fig 3** as well as **Fig 2** of the revised manuscript. These bars denote 1 standard error of the mean and were calculated by dividing the standard deviation by the square root of the numbers of pairs of SNPs in the particular bin.

The bins on the x-axis of **Fig 3A** of the revised manuscript were chosen to have quantiles with approximately equal numbers of SNP pairs in each bin.

Here we binned the data into 6 quantiles using the R package binr. The first quantile has more pairs than the others (because many of the SNP pairs have very small recombination distances from each other), but the others are similar. See below for the number of pairs in each bin.

[0, 1.1960e-07) 3502 Pairs
[1.1960e-07, 4.9961e-05) 700 Pairs
[4.9961e-05, 0.0004) 701 Pairs
[0.0004, 0.0020) 700 Pairs
[0.0020, 0.0082) 700 Pairs
[0.0082, 0.2876] 701 Pairs

Now the tick marks are round numbers and the scale is linear. See below for the revised version of **Fig3A**.

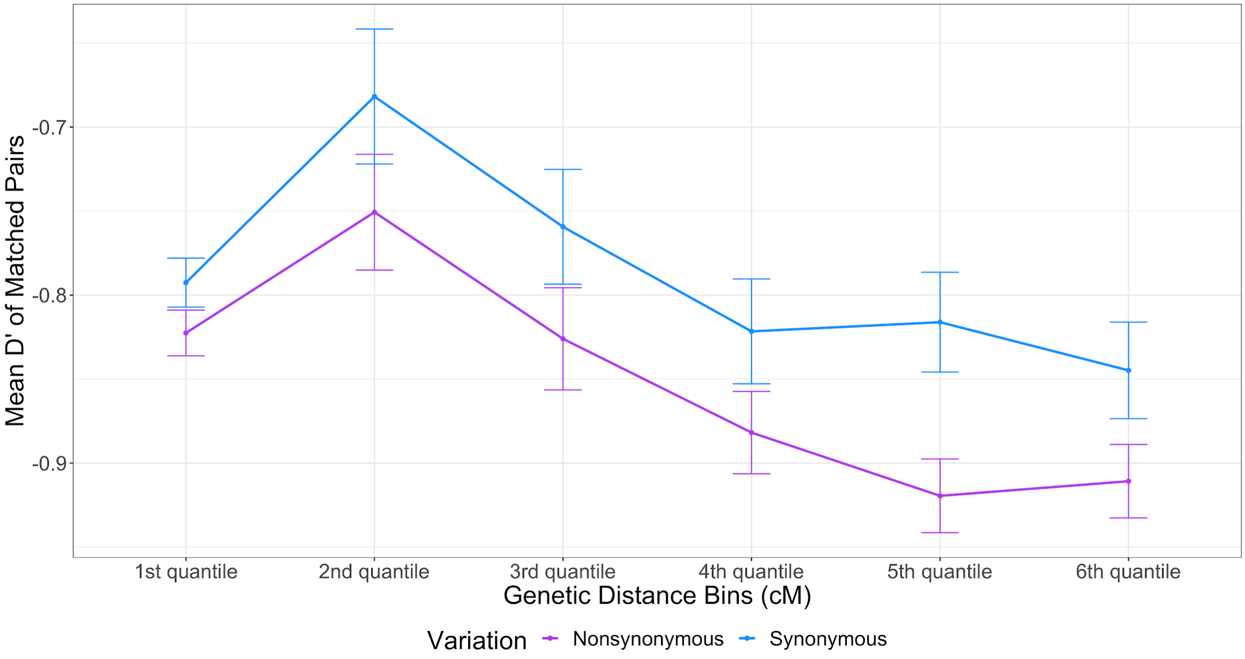

10. Figure 4b presents traditional LD metrics, seeming to contradict the statement on p. 18 that they cannot be computed on real data due to lack of phased information. Are these calculated using computational phasing? Are there any limitations to this approach (e.g. that it cannot be applied to variants below some MAF threshold)?

We apologize for the confusion on this point in the previous version of the manuscript. Fig 4B of the previous version of the manuscript (**Fig 3B of** the revised manuscript) shows traditional LD statistics computed from Phase 3 of the 1000 Genomes Project data. The 1000 Genomes Project used imputation approaches to computationally phase the data. Thus, we were able to compute the LD statistics after computational phasing.

However, as it's possible that this phasing approach introduced errors, particularly at low-frequency variants, and relied on LD to perform the imputation, we wanted to replicate our results in non-computationally phased, non-imputed data. To do this we turned to the higher coverage version of the 1000 Genomes data produced by the New York Genome Center (presented in **Fig 5C and 5D** of the

revised manuscript) that did not include computational phasing. Thus, for these samples, we only used the H_R^j statistic.

We now clarify this point in the revised manuscript where we write, (lines 284-296) "The standard LD statistics r^2 , D, and D' we used are based on counts of the number of haplotypes that contain both derived alleles at both loci (p_{AB}), which require phased haplotype information. Often when studying nonmodel organisms or unprocessed genotype data we typically do not have this information and would have to rely on computational phasing. The performance of computational methods to impute or phase variants becomes more challenging for rare variants [47,48], which are the focus of our study. Further, when studying sites influenced directly by natural selection, the assumption of HWE may be violated and the correlation between the diploid allele counts (as denoted by {0,1,or 2}) may not equal the correlation between the haplotypes for two markers.

Here we introduce new statistics that can be directly computed from diploid genotype data to quantify whether derived alleles are more likely to be coupled (i.e. on the same haplotype) or in repulsion (i.e. on different haplotypes) for different functional annotations."

Reviewer #2: In their manuscript "Negative linkage disequilibrium between amino acid changing variants reveals interference among deleterious mutations in the human genome" Garcia and Lohmueller examine the signal of LD between deleterious alleles. The study first analyses the expected patterns of LD using forward in time simulations, before studying these patterns in empirical human population data. The study shows that LD between deleterious loci is lower than neutral equivalents, suggesting interference. In addition, the authors introduce a new statistics (H_R) that allows to detect interference in unphased genomic data.

Overall, this is a very well conceived study. In nicely combines intuition building through simulations with empirical analyses. The manuscript is timely, well-written and interesting. Few modifications and additional information would make the manuscript more accessible to a broader audience.

Thank you for these comments and for appreciating the significance of our work.

- I find it quite difficult to wrap my head around negative LD when values in the figures (e.g., Fig. 1 and 2) are positive. I think it would increase the accessibility of the manuscript present values of D' instead of r^2.

We agree with the reviewer that it is difficult to study negative LD when the values in the figures are positive. In response to comments from Reviewer 1, we have moved Figure 1 from the previous version of the manuscript into SI (now **S2 Fig** of the revised manuscript). For the new **Fig 1** (which only includes nonsynonymous doubletons), we now present D' values, as suggested by the reviewer. Further, for the other main text figures that show traditional LD statistics (e.g. **Figs 3, 4)** we also present D' values as well. For several of the SI figures, we still show r^2 because it is commonly used by human geneticists in GWAS and we think it is valuable to show how it is also affected by negative selection. Incidentally, the

D' decay plots would not necessarily be negative for **SI Fig 2** of the revised manuscript (Fig 1 of the previous version of the manuscript). We only see negative values of D' when analyzing frequency-matched low-frequency alleles (from 1-5%, see **S1 Table**).

Lastly, we have tried to clarify in the text of the manuscript that "excess of negative LD" means (line 109) seeing fewer pairs of variants in positive LD which means that variants are less likely to occur on the same haplotype than expected under independence. For example, on lines 109-111 of the revised manuscript, we state, "In our study, an excess of negative LD means the same thing as seeing fewer pairs of variants in positive LD, which shows that variants are less likely to occur on the same haplotype than expected under independence."

- It would be good to see results of LD for higher frequency alleles. Do they show the same pattern as doubletons, or does it change? Some deleterious alleles might actually be at higher frequencies due to demography or introgression. Would be nice to see if there is a threshold for the observed pattern.

Thank you for this suggestion. We have now investigated LD patterns for higher frequency alleles than doubletons in simulated data. They show a similar pattern as doubletons until the frequency of variants gets higher than about 5/100 or 5%. This can be found as **S1 Table**.

- How is the LD calculation and pattern influenced by the difference in diversity between NS and S loci? Did you compare only pairs with equal physical distance?

These comparisons are made for pairs of frequency matched variants. That is to say, that each pair of NS variants at a particular frequency (e.g. a doubleton) was matched to a pair of S variants at that same frequency. Therefore, the presence or absence of diversity or differences in allele frequencies are not expected to influence the difference in LD between NS and S loci seen in our study. We also matched the physical distances between pairs of NS and S SNPs. We compared only pairs with equal physical distance (within 50 bp). The efficacy of our matching procedure is illustrated in **S13 Fig** of the revised manuscript. We also better clarify our matching procedure in the revised manuscript on lines 328-333 where we write "for each pair of NS SNPs, we extracted a pair of S variants with a similar physical distance between variants (bp), the same allele count between pairs, a similar genetic distance (cM), similar mean *B*-value amongst pairs of variants, and located on the same chromosome as the NS pair. With these matching criteria we compare pairs of variants that have similar levels of diversity and physical distance between variants. **S13 Fig** shows how closely we required the pair of S SNPs to match the pair of NS SNPs across these characteristics."

- The use of H comes a bit out for the blue for me and could maybe be better integrated into the flow. It would be particularly useful the show correlations of other LD measures with H. The fact that you have phased data should not harm H calculations and how does this compare to unphased results?

Thank you for bringing this to our attention. In the revised manuscript, we tried to integrate the $H_R^{(j)}$ statistics better in the main text. First, we have changed the structure of the manuscript to describe the

$H_R^{(j)}$ statistic after we present some simulations using traditional LD-based statistics (as suggested by Reviewer 1). Second, we better motivate the $H_R^{(j)}$ statistics by stating (lines 284-296), “The standard LD statistics r^2, D, and D' are based on counts of the number of haplotypes that contain both derived alleles at both loci (p_{AB}), which require phased haplotype information. Often when studying nonmodel organisms or unprocessed genotype data we typically do not have this information and would have to rely on computational phasing. The performance of computational methods to impute or phase variants becomes more challenging for rare variants [47,48], which are the focus of our study. Further, when studying sites influenced directly by natural selection, the assumption of HWE may be violated and the correlation between the diploid allele counts (as denoted by {0,1,or 2}) may not equal the correlation between the haplotypes for two markers. Here we introduce new statistics that can be directly computed from diploid genotype data to quantify whether derived alleles are more likely to be coupled (i.e. on the same haplotype) or in repulsion (i.e. on different haplotypes) for different functional annotations.” at the start of this section of the manuscript.

The reviewer is correct that the phased and unphased values for $H_R^{(j)}$ are identical to each other. Additionally, we created a new figure (S11 Fig of the revised manuscript) showing the correlations between H_R^1 , H_R^2 and D'. We also computed Spearman’s correlation coefficient of the summary statistics in 1,500 bp windows. For both singletons and doubletons, our $H_R^{(j)}$ statistic is highly correlated with D' ($\rho > 0.945$ (p-value < 2.2e-16)).

Revised Text

(lines 321-324) Additionally we computed Spearman’s correlation coefficient (ρ) between mean H_R^2 and mean D' as well as mean H_R^1 and mean D' (**S11 Fig**). In all cases, the $H_R^{(j)}$ statistics are highly correlated with D’ ($\rho > 0.945$, p-value < 2.2e-16). Thus, we conclude that $H_R^{(j)}$ can detect interference between deleterious variants using unphased genotypes.

- How is the nomenclature for H_R determined? Not sure I like the use of "H" in this context as it is used already extensively (Not that many other letters are being used uniquely). If you want to stick to "H" maybe it would be good to include LD as index instead of R to make clear that it's a measure of LD.

We use the letter R as the index because $H_R^{(j)}$ is a measure of ‘repulsion’ in a data set. ‘R’ is used instead of “LD” because R is one letter. We tried to use ‘l’ here but it looks too much like an I or the number one. We added clarification about the nomenclature of $H_R^{(j)}$ by giving two examples and added the meaning of the R to make it clear that it’s a measure of repulsion.

Specifically we write, (lines 308-313) “Applying this statistic to only doubletons in a sample of 50 diploid individuals, corresponding to variants with frequency of 2/100, we call it $H_R^{(2)}$. Essentially, $\mathbf{H_R^{(j)}}$ counts the average number of individuals heterozygous at both SNPs for pairs of SNPs at a given distance apart from each other. This statistic was created to measure the amount of repulsion in a sample, and that is what the subscript “R” stands for.”

- I was wondering how many sites are actually used for each analysis in the empirical data. Could you maybe include a supplementary table that indicates the number of sites used, im particular for the H_R analysis.

Thank you for suggesting that we clarify this point. We created **S2 Table** with the number of pairs of variants in each empirical based analysis. For **Fig 4b** there is a table that includes the number of variants for each analysis. For the $\mathbf{H_R^{(j)}}$ analysis in **Fig 6,** we included the number of pairwise comparisons in the figure legend. **S2 Table** is shown below:

Figure	Number of pairs	Matching between S and NS pairs*	Analysis	Notes
Fig 3	7,004	Yes	Variants with allele count 1-5	Same variants as Fig S11 (Low Coverage 1KGP)
Fig 4b	CEU =2,160; CHB = 2,810; JPT = 1,652; MXL = 5,926; YRI = 7,004	Yes	Variants with allele count 1-5	Low Coverage 1KGP
Fig 5a	35,308	No	Variants with allele count 2	Low Coverage 1KGP
Fig 5b	245,193	No	Variants with allele count 1	Low Coverage 1KGP
Fig 5c	33,273	No	Variants with allele count 2	High Coverage NYGC
Fig 5d	231,840	No	Variants with allele count 1	High Coverage NYGC
Fig 6	6,066	Yes	Variants with allele count 1-5	Pairs with 0 cM were removed (Low Coverage 1KGP)
SI Fig 11	7,004	Yes	Variants with allele count 1-5	Same variants as Fig 3 (Low Coverage 1KGP)

- While I think a uniform s is a good start and helps to build intuition about the effect, it would be great to have one or two additional simulations with (e.g., gamma) distributions that would allow to track which combination of effect sizes create the pattern. Or at least explore this further in the DFE simulations. What combinations of s create negative LD and which ones not. This would be useful for the interpretation of empirical data.

We thank the reviewer for this suggestion. We include simulations using a gamma DFE for deleterious mutations in **Fig 1** of the revised manuscript as well as in **S2, S5, S6, and S7 Figs**. In terms of when one expects to see the HRI effect, the impact on LD occurs with intermediate strengths of selection. For very strong selection, deleterious mutations are effectively removed from the population and are not segregating at appreciable frequency. Thus, here there is no interference or generation of negative LD (see the comments from Reviewer 3 on this topic as well). As variants become neutral, the effect of selection on LD dissipates. **Fig 1** shows that the gamma DFE behaves similarly to variants with *s*=-0.001. Thus, this result suggests that HRI is expected to have an impact on nonsynonymous variants in human datasets.

To explore an additional DFE, we simulated 2,800 simulation replicates with a constant recombination rate (*r*=1e-08) under Model 1 with NS mutations coming from a gamma DFE with shape parameter 0.186 and E[*s*] =-0.00657402090856. Here all NS mutations had a dominance coefficient of *h*=0.5, and the parameters of this gamma defined DFE were also inferred by Kim et. al 2017. This DFE is different than the one described in the paper (shape parameter 0.186 and expected selection coefficient E[*s*] = -0.01314833). Similar to **S6 Fig**, we see that nonsynonymous doubletons have lower values of *D* than synonymous doubletons for certain distances. As the distance between pairs increases, synonymous and nonsynonymous doubletons are predicted to have similar *D* values. Since this decay curve is so similar to the one in **S6 Fig 6** we did not include the figure below in the manuscript.

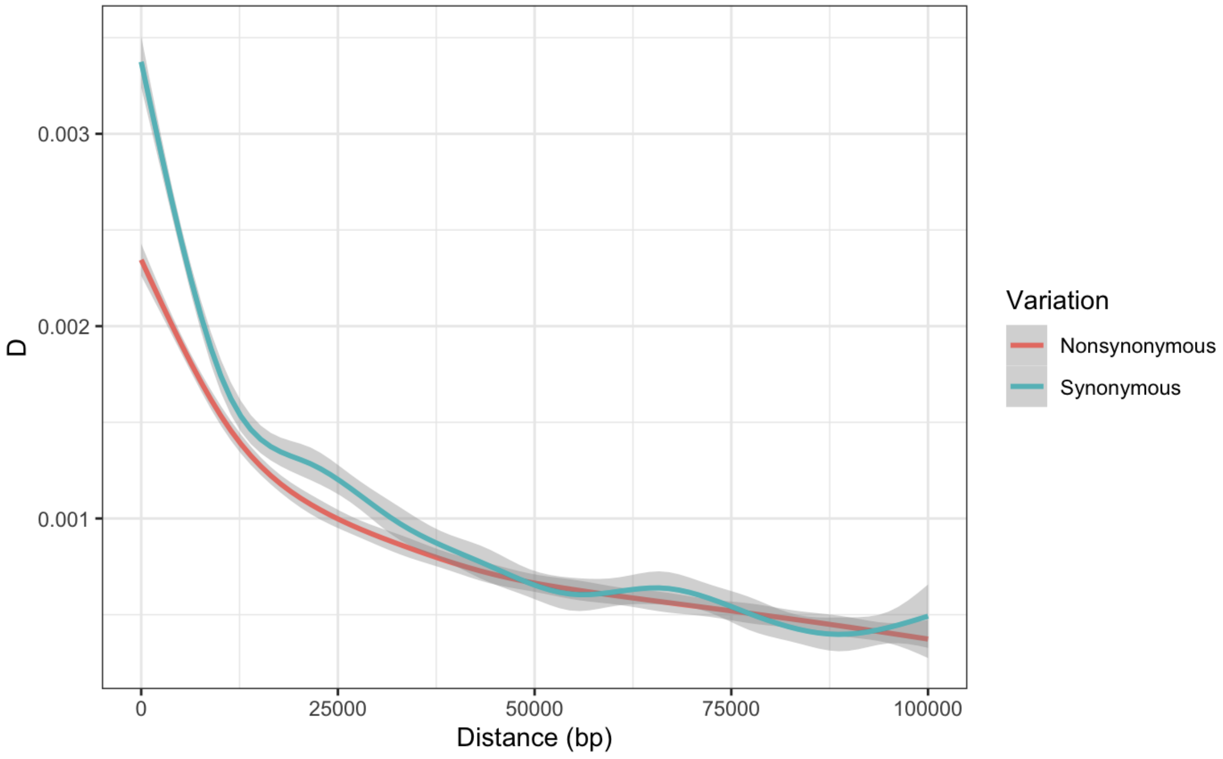

It is also important to note that the dominance coefficient also has a substantial effect on the LD patterns (**Fig 1**).

We discuss the dependence of LD statistics on the selection and recombination parameters in the revised manuscript where we write, (lines 467-477) "While we use simulations to quantify the effects of interference on LD statistics, a limitation of this study is that we do not simulate full human genomes and directly assess whether the model parameters can fully explain the empirical data. The shape and scale of the DFE that we simulated under closely recapitulates empirically observed site frequency spectrum in human genetic variation data [40]. However, for tractability, we did not incorporate empirically measured recombination maps into our simulations, and instead simulated under uniform recombination rates. Because of this, the LD decay curves in our sample may not quantitatively match the curves seen in empirical data. With these limitations in mind, we compared trends from these simulations qualitatively to our empirical data. Future work can test whether realistic genetic maps, functional annotations, and a DFE can match genome-wide patterns of LD. " We also write (lines 271-274) "Both the simulations with selection coefficients of NS mutations coming from a gamma distributed DFE and simulations with NS mutations with constant selection coefficients predict deleterious doubletons should have lower values of mean *D'* relative to neutral doubletons."

- It would be useful to introduce the concept of negative LD in the introduction

We agree and define the concept of positive and negative LD throughout the Introduction. Below we show an example.

Revised Text

(lines 70-74) If two variants are in linkage disequilibrium, they can either be in positive LD or negative LD. Positive LD occurs when derived alleles appear on the same haplotype more often than expected under independence (i.e. linkage equilibrium) and negative LD occurs when derived alleles appear on the same haplotype less often than expected under linkage equilibrium.

- "... or negative synergistic epistasis play a pervasive role in shaping patterns of LD between proximal NS variants in the human genome." This seems to imply that you tested for negative synergistic epistasis, but this is not the case. I would suggest to rewrite.

The reviewer is correct that we did not test for negative synergistic epistasis. However, we cannot rule out the effects of negative epistasis on the empirical LD patterns either. We show that HRI effects (without negative synergistic epistasis) can generate patterns like those seen empirically.

Revised Text

(lines 187-189) Our findings of an excess of negative LD between pairs of putatively deleterious variants suggest that interference might play a pervasive role in shaping patterns of LD between proximal NS variants in the human genome.

- Would be good to give more details on the simulations in the M&M section. Even if some parameter values (N and generations) are mentioned in the Results, I think it wouldn't harm to have them all together in the M&M section. This will make the workflow more reproducible.

We added more details on the simulations in the Methods section of the revised manuscript. Additionally, we added simulation scripts and data to this paper's github repository (https://github.com/JesseGarcia562/garcia_and_lohmueller_2020).

- Does NS and S come from "synonymous" and "non-synonymous"? Does this not unnecessarily narrow the sites. The simulations are not limited to those and in reality other loci might be as deleterious (e.g., promotors). I think it would be clearer to use deleterious and neutral throughout the manuscript. In the empirical part the use of non-synonymous as proxy for deleterious can be specified.

NS and S do refer to synonymous and nonsynonymous variants. We attempted to make this more clear in the text. Yes, intergenic deleterious variation exists, however, in our simulations we limit ourselves to only studying "exonic" variation. For our study, this does not include loci such as promoters which are upstream of transcription start sites. We chose to keep nonsynonymous and synonymous because these terms refer to the precise types of variants analyzed in our study. To characterize them as neutral or deleterious would require some additional assumptions that one may not be willing to make. For example, some nonsynonymous mutations in the empirical data may be nearly neutral in simulations with a gamma distribution of fitness effects. However, we now state early in the Results that nonsynonymous mutations are thought to be deleterious and synonymous are more neutral.

Revised Text

(lines 195-198) The rate at which deleterious NS mutations arise in our simulations is for every 1 synonymous (S) mutation there are 2.31 NS mutations. This value is derived from degeneracy of the codon table of eukaryotes [40]. We refer to mutations as NS and S throughout this study because these terms refer to the precise types of mutation analyzed in empirical data.

Reviewer #3: In this manuscript, the authors use simulations to study how patterns of linkage disequilibrium (LD) are affected by negative selection. They show using simulations that deleterious mutations can be brought into negative linkage disequilibrium by the action of linked selection, i.e. even in the absence of epistatic interactions between the mutations. They show that the excess of negative linkage disequilibrium can be maintained in complex demographic models thought to be realistic for humans, and also demonstrate that an excess of negative linkage disequilibrium can be observed in empirical data in the difference in patterns of LD between nonsynonymous and synonymous variants. They highlight that an excess of negative LD due to linked selection can lead to problematic estimates of quantities inferred from genome-wide LD patterns under the assumption that these are not significantly impacted by linked selection. I think this is an important and valuable point, especially given the central role that these methods play in statistical and population genetics.

We thank the reviewer for these positive comments on our study.

- is there an implementation code for H_R that other people could use on their data? Would be great if you could implement it to work on VCF files for others to test those patterns. I think this would increase the impact of your study substantially.

This is a great suggestion. We included an R script to compute $H_R^{(j)}$ on SLIM VCFs here:

https://github.com/JesseGarcia562/garcia_and_lohmueller_2020/blob/master/computing_hrj_slim.R

And with their own data sets here:

https://github.com/JesseGarcia562/garcia_and_lohmueller_2020/blob/master/compute_hrj/compute_hrj.R

Fig. 1 why do strongly deleterious alleles show higher LD than neutral alleles? if it was only for the lack of interference as described in the "results" would we then not expect the no difference to neutral. But observed is an increase. Can you give further explanations for this increase?

Figure 1 of the previous version of the manuscript (now **S2 Fig**) shows the LD decay for all variants (i.e. both synonymous and nonsynonymous), not only the deleterious ones. Further, we included all allele frequencies in this analysis. Thus, differences in the proportion of neutral vs. deleterious alleles

segregating as well as shifts in the frequency spectrum caused by selection are likely to account for this result. The fact that it is hard to develop intuition about how selection affects LD from a plot like this is why we focus subsequent analyses on matching for allele frequencies and functional annotation, as they are easier to understand the biological basis. **S2 Fig** is included for completeness to provide an overview to suggest that selection can perturb LD in complex ways.

Revised Text

(**S1 Text**) This is likely in part due to the fact that strongly deleterious mutations are not expected to segregate within a population for long. Therefore, the effects of interference are diminished [1]. However, it is still possible to observe the consequences of background selection from these deleterious mutations on overall patterns of LD. Zeng and Charlesworth have previously shown that LD can be elevated by background selection to an extent that is well summarized by a change in effective population size [2]. We believe this in concordance with our simulations here.

Overall, I find the manuscript very interesting and the main conclusions convincing. I believe it represents a valuable contribution to the literature, but I have one substantial concern, which is that I don't believe that the effect that leads to an excess of negative LD has been accurately described and interpreted. Specifically, there is existing theoretical literature on Hill-Robertson interference causing negative LD that explains why and when Hill-Robertson interference causes negative LD, both in cases of positive selection in asexually evolving populations (see Gomez et al. 2019 Genetics) and negative selection in both asexually evolving and recombining populations (see Good et al. 2014 PLoS Genetics). The latter work in particular shows that negative LD may or may not arise in the presence of linked negative selection, depending on how efficient selection is at purging deleterious mutations from the population. It would be important for the text of the manuscript to be revised to accurately reflect this effect (and to acknowledge these earlier authors describing the effect) — I give details in my first major comment.

By considering Fisher's fundamental theorem, one can show that as long as selection is able to efficiently remove this load and Muller's ratchet is operating slowly compared to the timescale of coalescence, excess negative LD is not expected as a result of linked selection. By Fisher's fundamental theorem, the rate of change in the mean fitness, v is related to the fitness variance sigma^2, mutation rate and selection coefficient s according to: v = sigma^2 - uLs. Here we see that the Poisson case of efficient selection described above corresponds to v = 0 and sigma^2 = u L s, whereas the ratcheting case has v < 0, and so sigma^2 < u L s, and deleterious mutations tend to be in negative LD. This effect persists in the presence of recombination — see Good et al. 2014 PLoS Genetics, and in particular Fig. S1, and the Supplementary Texts.

We thank the reviewer for this detailed explanation as well as suggesting these important references. We agree and have significantly revised the manuscript when describing how and when HRI effects are expected to occur. We added citations and context to Good [23] and Gomez [24] to our paper. We also emphasized the importance of genetic drift and variance in fitness across linkage blocks for interference and excess negative LD.

Revised Text

(lines 102-204) If, however, deleterious mutations occur on different haplotypes in a population, then the variance in fitness across haplotypes is reduced, as they all carry a similar number of deleterious alleles [23,24].

(lines 112-117) Specifically, by considering Fisher's fundamental theorem, as long as selection is able to efficiently remove load and Muller's ratchet is operating slowly compared to the timescale of coalescence, excess negative LD is not expected as a result of linked selection [23]. For instance, a population with a single non-recombining chromosome with deleterious mutations that are removed at the same rate at which they are created (in mutation-selection balance), does not experience HRI [23,28].

(lines 147-152) Recent literature on Hill-Robertson interference proposes mechanistically why and when Hill-Robertson interference causes negative LD, both in cases of positive selection in asexually evolving populations [24] and negative selection in asexually evolving and recombining populations [23]. In these works, the authors study how the variance in fitness within "effectively asexual linkage blocks" is important when interference is prevalent.

1) The argument outlined in the paragraph starting on the bottom of page 5 and continuing on page 6 is not entirely accurate. It attributes the entire effect of negative LD to an increase in relative fitness of single mutants due to the presence of multiple mutants. Yet it is not difficult to show that this effect on its own does not lead to an excess of negative LD. Consider for instance a population with a single non-recombining chromosome in mutation-selection balance, in which deleterious mutations are removed at the same rate at which they are created, so that the population is not experiencing Muller's ratchet. For simplicity, let's focus on the case that all mutations have the same effect on fitness, -s, though this is not essential to the argument. In this case, at mutation-selection balance, the population assumes a Poisson-shaped fitness distribution with an average uL/s mutations per individual, where u is the per-site mutation rate, and L the number of sites, as described by Haigh (1978) and others. This means that the deleterious mutations are distributed independently, as if they were freely recombining and excess negative LD does not arise in this case. This is in direct contrast to the explanation given by the authors. Note that this rationale also agrees quite well with the simulation results seen here — there is an intermediate strength of selection at which excess negative LD is strongest, which also agrees with expectations of the rate of Muller's ratchet being maximized at intermediate fitnesses. However, it does suggest that many of the simple arguments offered here do not stand up to scrutiny.

We agree with this explanation and thank the reviewer for pushing us to be more precise in our explanation of HRI. We have revised this section of the manuscript extensively to include the points made by the reviewer. Specifically, we now clarify that HRI can occur in finite populations experiencing drift which are not in mutation-selection balance. In mutation-selection balance, there will not be an excess of negative LD.

Revised Text

(lines 111-117) Importantly, population genetic models without drift do not predict an excess of negative LD. Specifically, by considering Fisher's fundamental theorem, as long as selection is able to efficiently remove load and Muller's ratchet is operating slowly compared to the timescale of coalescence, excess negative LD is not expected as a result of linked selection [23]. For instance, a population with a single non-recombining chromosome with deleterious mutations that are removed at the same rate at which they are created (in mutation-selection balance), does not experience HRI [23,28].

2) The focus on doubletons to quantify the effects of negative selection is not well justified from a theoretical standpoint (though I agree that using doubletons appears generally better than the common practice of using singletons, as the authors explain). Ultimately, the important population-genetic factor is the frequency of an allele in the population, rather than in the sample, and depending on the sample size, doubletons can represent vastly different frequencies. In many modern datasets, singletons and doubletons can be at such low frequencies they are primarily affected by genetic drift for reasonable strengths of selection, which means that the main effect that the researchers aim to study is likely difficult to observe on theoretical grounds, and possibly attributable to other causes. In general, while I do agree with the authors that patterns of LD are strongly influenced by frequency, the choice of target frequency should be guided by theoretical considerations and parameter values, in addition to heuristics for the elimination of technical artifacts in a dataset (which I agree are also very important).

For instance, in some cases examined here — for instance for s = 0.001, N = 10 000, Ns = 10, which means that all variants below a frequency of 1/Ns = 10% are primarily affected by drift. Doubletons in these samples (n= 100) are expected to be present in the population at frequencies 2% or lower, meaning that on their own, they should not be strongly impacted by selection. In fact, for these parameters, we can quite confidently claim that the effects of negative selection should be very weak at the target frequencies if all haplotypes contain no more than two deleterious mutants. Thus, any effect visible at these frequencies must be attributable to statistical features stemming from linkage to (large numbers of) other deleterious mutations, which reinforces the point above that the causes of negative LD are subtler than described here.

These are good points. For the purposes of both the simulation and empirical analyses, we focus on a sample size of 50 diploid individuals. We agree that the selection coefficients of doubletons in a sample size of 50 likely have different selective effects than doubletons in larger sample sizes. We have added some additional text to the revised manuscript clarifying that allele frequencies other than doubletons might be better for much larger sample sizes (lines 477-481) "We focused on variants with minor allele count less than five because in our sample size of 50 they are affected by selection **S3 Fig**. In many modern datasets, sample sizes are much larger and have many more than 50 individuals. In such datasets singletons and doubletons can be at very low population allele frequencies. In these datasets it may be better to focus on other allele frequency categories for detecting HRI. " Based on our simulations, doubletons and other low frequency variants in a sample size of 50 show an impact on LD patterns and we agree this has to do with many selected sites in a region.

Our rationale for focusing on sample frequency rather than population frequency is that we typically only have a sample from the population. Thus, the sample-based LD statistics are what we can directly observe and are most relevant.

For the matched-pairs permutation test using *D′*, we use variants with allele counts of 1-5 (taking care to match the frequencies between NS and S pairs). Thus, we do not exclusively focus on doubletons for this analysis.

Additionally, we added **S1 Table** to the revised which looks at mean *D′* across other allele frequencies in simulations. This table suggests that NS doubletons have lower mean *D′* than S doubletons and that the lower mean for NS variants persists for variants with equal to and less than 5% frequency.

Lastly, we provide additional rationale for focusing on doubletons in the revised manuscript. We write, (lines 222-224) "From a theoretical perspective, studying doubletons is advantageous because of their abundance in a sample from a population as they are the most common type of variants after singletons under the standard neutral model [45]."

To avoid these technical artifacts of sample size, and understand better the frequency-dependence of the effect describes here, would it be feasible to look at these statistics as a function of the entire frequency spectrum and attempt to identify transition points in ways that are self-consistent with the input parameters?

S1 Table shows *D*' as a function of discrete allele frequencies in simulations while conditioning on population demography and sample size. We see that as the frequency of variants increases deleterious NS variants no longer display lower levels of LD relative to neutral S pairs of variants. We see that the differences in mean *D*' between NS and S pairs decreases as allele frequencies increase. After allele frequencies increase past 5%, the HRI effects appear to no longer be as pronounced.

1) I would argue that the statement that there has been comparatively less empirical work quantifying Hill-Robertson interference as a bit ungenerous — given how broadly the term is applied, one could argue that many papers attempting to quantify the strength of background selection (including some classical ones by Charlesworth et. al, as well as more recent work by e.g. McVicker et al (2009) PLoS Genetics, Elyashiv et al. (2016) PLoS Genetics) are doing precisely that. Perhaps make the statement a bit narrower to clarify?

We completely agree and removed the statement that there "has been comparatively less empirical work quantifying HIll-Robertson interference" from the Introduction of the revised manuscript.

2) I think it is a great point that unmodeled negative selection may contribute to differences between empirically observed LD patterns and demographic models fit to the site frequency spectrum (page 26, Discussion). However, I disagree with the suggestion that filtering variants that may be affected by selection should be sufficient, unless nearby neutral SNPs are also filtered. As this manuscript (and

others) demonstrate, linkage between deleterious variants is critical to producing negative LD, and this form of linkage also affects patterns of variation at neutral sites.

We agree with the reviewer’s assessment. We now recommend carefully filtering variants that may be affected indirectly (linked selection, interference, background selection) or directly by selection by using functional annotations and recombination rate information.

<u>Revised Text</u>

(lines 503-506) Unmodeled negative selection may contribute to this lack of fit. We recommend carefully filtering regions of the genome that may be affected indirectly (linked selection, interference, background selection) or directly by selection by removing loci that have a short recombination distance to functionally annotated loci [56,57].
