# Supplementary material for: Negative linkage disequilibrium between amino acid changing variants reveals interference among deleterious mutations in the human genome
Source: PLoS Genet. 2021 Jul 28;17(7):e1009676. doi: 10.1371/journal.pgen.1009676 (PMC8351996; doi:10.1371/journal.pgen.1009676)
Supplement: Attachment — Submitted filename: Second_Response_to_Reviewers_2021.v3.pdf [file pgen.1009676.s021.pdf]

Response to Reviewers

Reviewers' comments are in Black: Authors' responses are in Blue

Reviewer #1: Thank you to the authors for their thorough response - I have no further comments.

Thank you for your previous comments, they have greatly improved the manuscript.

Reviewer #2: The authors have made a great effort to address my comments and improved their manuscript.
I have no further comments.

Thank you for critically reading the manuscript and suggesting substantial improvements.

Reviewer #3: The authors have done a commendable job in addressing my concerns, and in my opinion have produced a very interesting piece of work. I would also like to thank them for formatting their revision so clearly and thus making it very easy to follow the changes in the revision process.

Thank you for your comments that helped improve and clarify parts of our manuscript.

I only have two further comments:

1) Figure 2 shows some pretty dramatic non-monotonicity in the mean H_R(1) and H_R(2) statistics in the simulated samples. This highlights a point that (as discussed by the authors with reference to real data) the effect size may be relatively subtle and somewhat noisy. Would it be possible to increase the sample size/number of simulations performed for Figure 2, so that the nonmonotonicity is ultimately averaged out, giving the reader a sense of what can be expected under ideal conditions. It is also good to see the effect of limited sampling (and I very much like how the authors accomplished to show both in Figure 6). I don't know whether this is computationally feasible at this point, but if at all possible, it would be very scientifically informative.

Thank you for this comment. In Figure 2 we increased the number of simulations from 1,000 simulation replicates to 4,300 simulation replicates. This did ultimately average out much of the non-monotonicity. We also included error bars which represent one standard error of the mean. The revised version of Fig. 2 is pasted below.

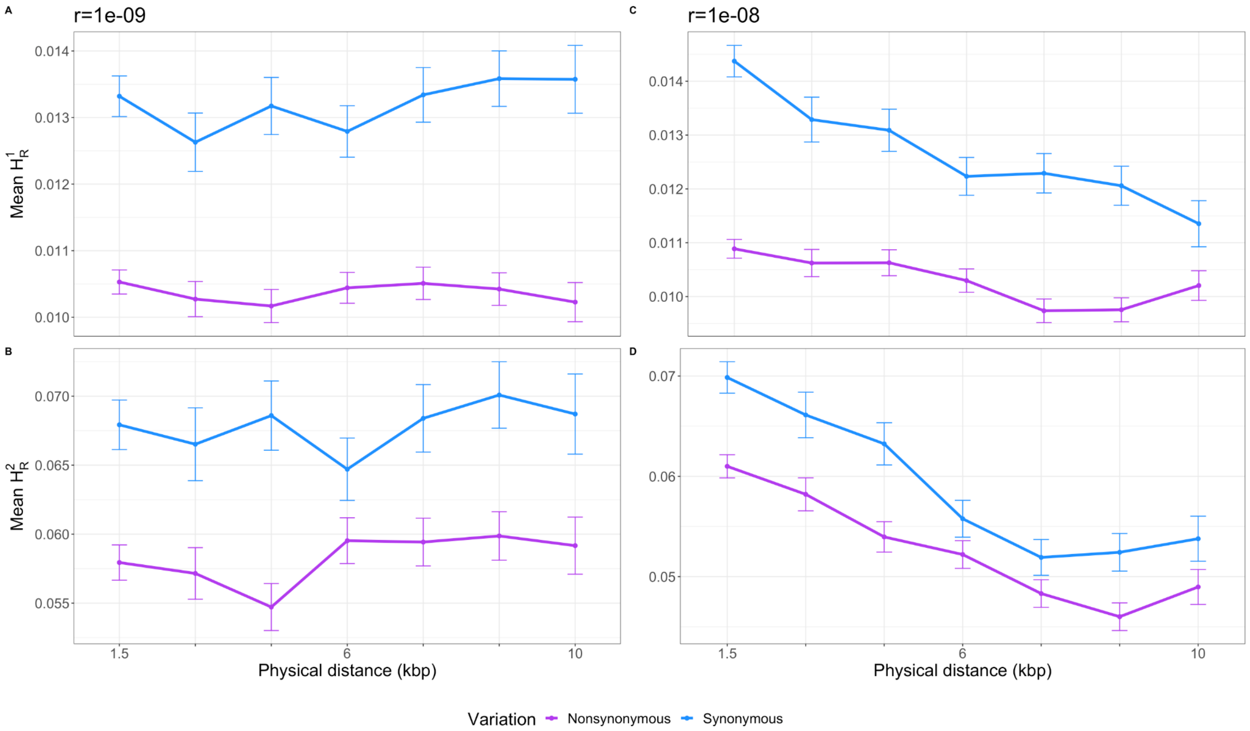

2) Lines 279, 280: “Our simulations predict that after deleterious variants increase above a frequency of 6% they will no longer have lower D’ values than neutral alleles.” — I recommend including a brief clause along the lines of “under the assumed simulation parameters” just to emphasize to the reader that this is not an absolute statement about a definitive cutoff in human genomes.

We agree with the reviewer’s comment. To emphasize that this is not an absolute statement, we have added “under the assumed simulation parameters” to the referenced sentence.

Revised Text

(lines 278-280) “Under the assumed simulation parameters, our simulations predict that after deleterious variants increase above a frequency of 6% they will no longer have lower D’ values than neutral alleles.”

Reference Updates

We updated four references during this revision. Reference 1 and 2 below were updated so that they referred to the peer-reviewed publication and not the biorXiv preprint. Reference 3 below was updated so that it included the editors and publisher of the book in which the cited chapter comes from. Lastly, we cited Reference 4 with two different citation numbers and they were condensed to 1 citation.

1. Anderson-Trocmé L, Farouni R, Bourgey M, Kamatani Y, Higasa K, Seo J-S, et al. Legacy data confounds genomics studies. Mol Biol Evol. 2020;37: 2-10.

2. Torres R, Stetter MG, Hernandez RD, Ross-Ibarra J. The temporal dynamics of background selection in nonequilibrium populations. Genetics. 2020;214: 1019–1030. doi:10.1534/genetics.119.302892
3. Felsenstein J. Sex and the Evolution of Recombination. In: Michod RE, Levin BR, editors. The Evolution of Sex: An Examination of Current Ideas. Sunderland, Mass., USA: Sinauer Associates; 1988. pp. 74–86.
4. Lewontin RC. The interaction of selection and linkage. I. general considerations; heterotic models. Genetics. 1964;49: 49–67.

We also fixed additional typographical and capitalization errors in the Bibliography. Please see the changes highlighted in the Track Changes. Lastly, we updated several of the Figure captions to better reflect the figures.
